# A Fungal Transcription Regulator of Vacuolar Function Modulates Candida albicans Interactions with Host Epithelial Cells

**DOI:** 10.1128/mBio.03020-21

**Published:** 2021-11-16

**Authors:** Philipp Reuter-Weissenberger, Juliane Meir, J. Christian Pérez

**Affiliations:** a Interdisciplinary Center for Clinical Research, University Hospital Würzburg, Würzburg, Germany; b Institute for Molecular Infection Biology, University Würzburg, Würzburg, Germany; c Department of Microbiology and Molecular Genetics, McGovern Medical School, The University of Texas Health Science Center at Houston, Houston, Texas, USA; Worcester Polytechnic Institute; Tel Aviv University

**Keywords:** *Candida albicans*, host-pathogen interactions, transcriptional regulation, vacuole

## Abstract

Microorganisms typically maintain cellular homeostasis despite facing large fluctuations in their surroundings. Microbes that reside on human mucosal surfaces may experience significant variations in nutrient and ion availability as well as pH. Whether the mechanisms employed by these microbial cells to sustain homeostasis directly impact on the interplay with the host’s mucosae remains unclear. Here, we report that the previously uncharacterized transcription regulator *ZCF8* in the human-associated yeast Candida albicans maintains vacuole homeostasis when the fungus faces fluctuations in nitrogen. Genome-wide identification of genes directly regulated by Zcf8p followed by fluorescence microscopy to define their subcellular localization uncovered the fungal vacuole as a top target of Zcf8p regulation. Deletion and overexpression of *ZCF8* resulted in alterations in vacuolar morphology and luminal pH and rendered the fungus resistant or susceptible to nigericin and brefeldin A, two drugs that impair vacuole and associated functions. Furthermore, we establish that the regulator modulates C. albicans attachment to epithelial cells in a manner that depends on the status of the fungal vacuole. Our findings, therefore, suggest that fungal vacuole physiology regulation is intrinsically linked to, and shapes to a significant extent, the physical interactions that *Candida* cells establish with mammalian mucosal surfaces.

## INTRODUCTION

Candida albicans is a prominent fungal species residing in humans. The majority of healthy adults carry this fungus as part of their normal gut microbiota (1, 2) although C. albicans can also thrive in other sites of the human body, including the oral cavity. Overgrowth of the fungus on the mouth’s mucosal surface causes a disease termed oropharyngeal candidiasis (OPC). As with other fungi, a debilitated immune system is the main risk factor associated with *Candida* infections. Indeed, OPC is one of the most prevalent manifestations in AIDS patients (3). In the last few years, the first unbiased and systematic searches for C. albicans genes involved in gut colonization and/or OPC have been reported (4–7). While a handful of the identified open reading frames (ORFs) could be linked to specific roles and cellular processes (based on previous studies in *Candida* or relying on the annotation of orthologs in the model yeast Saccharomyces cerevisiae), most of the “hits” derived from these genetic screenings remain uncharacterized. Investigating how this subset of uncharacterized *Candida* ORFs contribute to gut colonization and/or OPC is likely to reveal novel aspects of the biology of the fungus in the mammalian host.

The C. albicans *ZCF8* gene encodes a putative zinc cluster transcription regulator with a narrow phylogenetic distribution (orthologous genes can be found only within the *Candida* CTG clade). Through genetic screenings, our laboratory found that the *zcf8* null mutant strain displays two striking opposing phenotypes in mice: (i) impaired gut colonization after oral gavage (4) and (ii) increased fungal load in tongues in an OPC model (5). The molecular basis of these phenotypes, however, remained unexplored. The only other report on *ZCF8* in the literature demonstrated a role for this gene in promoting C. albicans adherence to silicone surfaces (8). In large-scale phenotypic screenings conducted under a variety of laboratory growth conditions, the C. albicans *zcf8* deletion mutant exhibited wild-type levels of proliferation across all conditions evaluated (9).

The fungal vacuole is a dynamic organelle with a range of functions, including the storage of amino acids, phosphate and calcium, glycoprotein turnover and hydrolysis, ion homeostasis, pH and osmotic regulation (reviewed in references 10 to 13). The vacuole lies at the center of fungal cell physiology, and as such, it is a highly responsive organelle that adjusts itself to both external and internal stimuli. Studies of S. cerevisiae indicate that the shape and number of vacuoles found in a cell often reflect a response to the surrounding environment. In proliferating cells, for example, high metabolic activity favors multiple medium-sized vacuoles, whereas nutrient limitation induces fusion events that result in one single organelle taking up most of the cell volume. A large size vacuole fits the needs of the cell to break down cellular material delivered to this organelle via autophagy and/or the multivesicular body pathway (11). Vacuole fission and fusion depend, at least in part, on the acidity inside the organelle which is typically maintained around pH 6.25 by the vacuolar ATPase. The activity of this proton pump generates the H^+^ ions that give the vacuole its characteristic acidic pH, which is critical for degradation processes, storage of metabolites, and energizing other vacuolar transporters (11, 14).

In this report, we investigate the role(s) that the *ZCF8* gene play(s) in the biology of C. albicans. Employing genome-wide approaches to systematically identify *ZCF8*-regulated genes, we establish that Zcf8p is a regulator of vacuolar function. In support of this notion, we found that deletion or overexpression of the regulator resulted in alterations of various vacuolar properties. We also demonstrate that *ZCF8* impinges upon the ability of the fungus to attach to mucosal surfaces and that the regulator’s effect on fungal adhesion depends, at least in part, on its regulation of the vacuole. Our results, therefore, establish a novel link between fungal attachment to mammalian host surfaces and the regulation of the fungal vacuole.

## RESULTS

### ChIP-Seq reveals that the transcription regulator Zcf8p binds upstream of genes encoding vacuolar and intracellular vesicle trafficking components.

The gene *ZCF8* encodes a putative zinc cluster transcription regulator. While genetic screenings have linked mutations in this gene to altered patterns of C. albicans colonization and infection in mouse models of gastrointestinal colonization (4) and oropharyngeal candidiasis (5), respectively, as well as to reduced adherence to silicone-covered surfaces (8), how Zcf8p effectuates these phenotypes remains unknown. Phylogenetic reconstruction based on the conserved DNA binding domain of the protein family in Hemiascomycetes (a group that includes the model yeast Saccharomyces cerevisiae as well as the entire *Candida* clade) suggests that this gene arose by gene duplication (or horizontal gene transfer from an unidentified source) relatively recently in evolutionary timescales because true orthologs could be identified only in the *Candida* or CTG clade (see Fig. S1 in the supplemental material).

10.1128/mBio.03020-21.1 FIG S1 Closest homologs of *ZCF8* in the *Candida* clade. (A) Cladogram depicting the phylogenetic relationships among extant species of the Candida and *Saccharomyces* clades. Colored arrows represent *ZCF8* and its closest homologs according to the *Candida* Genome Database and BLAST searches conducted with either full-length or conserved DNA binding domain protein sequences. Gene orthology assignments (same colors) follow independent reconstructions generated by the *Candida* Gene Order Browser. *OAF1* and *HAP1* (striped lines) are retrieved as “best hits” when queried with *ZCF34* and *ZCF5* sequences, respectively, in S. cerevisiae (however, neither *OAF1* nor *HAP1* are considered true orthologs of the indicated *Candida* genes). (B) Protein sequence alignment of putative DNA binding domains of *ZCF8* and its closest homologs. Alignment was performed by MUSCLE. Residues highlighted in red are the six cysteines characteristic of the zinc cluster domain. (C) Maximum likelihood phylogeny of *ZCF8* and its closest homologs. Alignments were generated using MAFFT (v.7.475). IQ-TREE 2 (v.2.1.2) was used to predict the best-fitting model (VT+F+R4), and FigTree (v1.4.4) (http://tree.bio.ed.ac.uk/software/figtree/) was used to generate the tree. Download FIG S1, TIF file, 0.5 MB. Copyright © 2021 Reuter-Weissenberger et al. 2021 Reuter-Weissenberger et al. https://creativecommons.org/licenses/by/4.0/ This content is distributed under the terms of the Creative Commons Attribution 4.0 International license.

**FIG 1:**
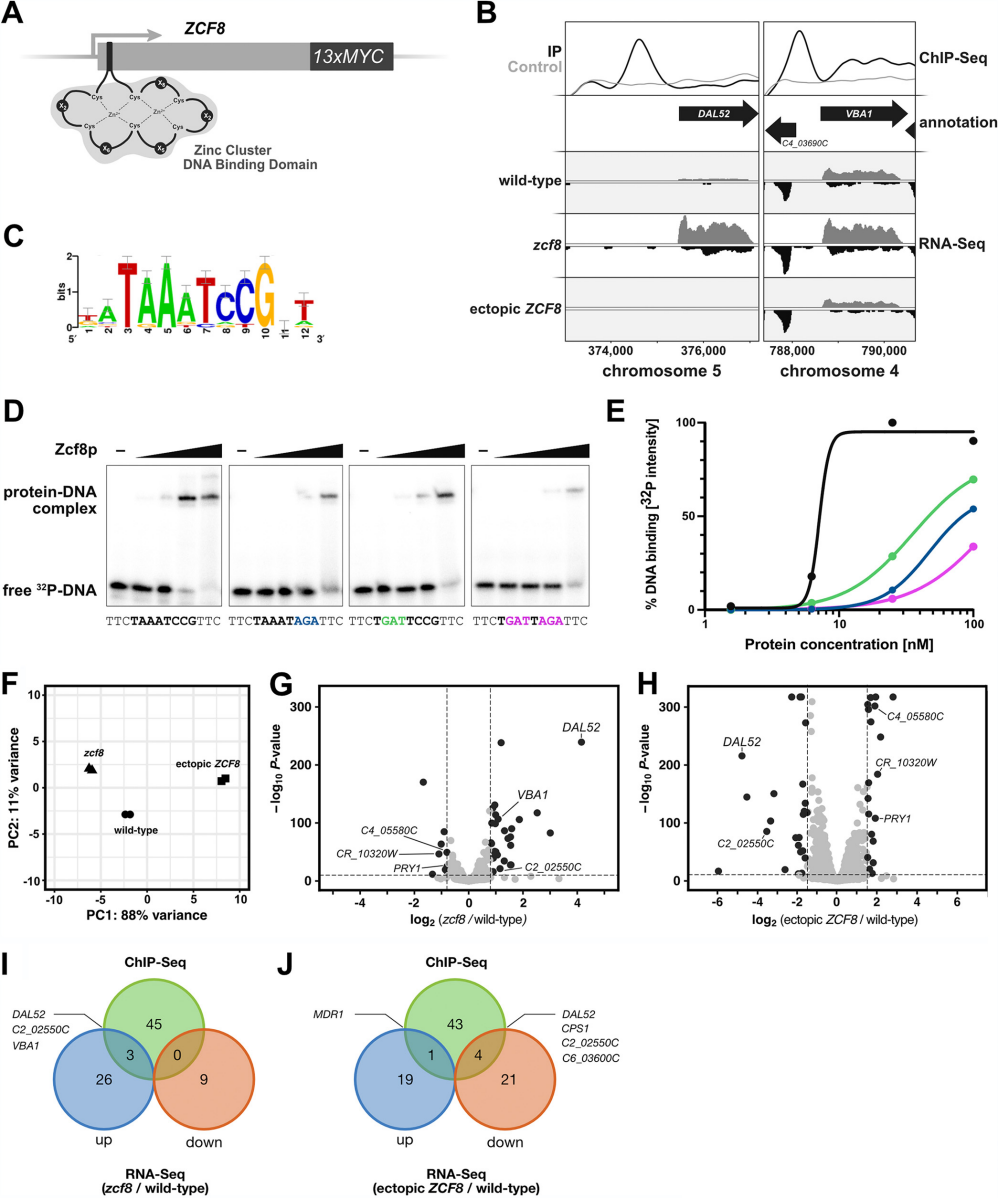
Identification of C. albicans genes controlled by the transcription regulator Zcf8p. (A) Schematic of the putative zinc cluster DNA binding domain found toward the 5′ end of the coding region of the C. albicans *ZCF8* gene. A sequence encoding the 13× MYC tag was introduced in frame at the 3′ end of the native *ZCF8* locus to facilitate immunoprecipitation. (B) ChIP-Seq and RNA-Seq tracks illustrating two genes directly regulated by Zcf8p. The top panel shows the IP (black) and control (gray) tracks resulting from ChIP-Seq. The bottom panels show the RNA-Seq tracks derived from three strains: wild-type strain, *zcf8* deletion mutant, and a strain overexpressing *ZCF8*. (C) DNA motif overrepresented in the top scoring regions occupied by Zcf8p according to the ChIP-Seq experiment. (D) Gel shift assays showing binding of the Zcf8 protein to its predicted binding site. ^32^P-labeled DNA fragments (0.4 nM) containing either the wild-type or mutant binding site were incubated with increasing concentrations (0, 1.56, 6.25, 25, and 100 nM) of the purified DNA binding domain (amino acids 57 to 158) of the Zcf8 protein for 30 min at room temperature in standard electrophoretic mobility shift assay (EMSA) buffer and resolved in 6% polyacrylamide gels run with 0.5× Tris-glycine-EDTA buffer (TGE; 10× TGE contains 30.3 g Tris HCl, 142 g glycine, 37.2 g EDTA per 1 L of solution). The DNA fragment evaluated (30 nt in size) is located upstream of the *DAL52* gene. Point mutations introduced at key positions are indicated in colors. (E) Quantification of the gel shifts shown in panel D. Colors correspond to DNA fragments with the wild-type sequence (black) or carrying point mutations introduced in different parts of the binding site (green, blue, and pink). Notice the log scale of the *x* axis. (F) Principal-component analysis (PCA) based on results of RNA-Seq experiment. Total RNA was prepared from the wild-type strain, *zcf8* deletion mutant, and a strain overexpressing *ZCF8*. All three strains were grown in defined medium lacking amino acids and ammonium sulfate as described in Materials and Methods. (G and H) Transcripts up- or downregulated in the *zcf8* deletion mutant (G) and in the strain overexpressing *ZCF8* (H). Shown are volcano plots where each dot represents one transcript. Significantly up- or downregulated transcripts are shown in black. (I and J) Venn diagrams showing the overlap between ChIP-Seq and the indicated RNA-Seq data sets.

To identify genes regulated by Zcf8p in C. albicans, we carried out chromatin immunoprecipitation followed by deep (Illumina) sequencing (ChIP-Seq). The native *ZCF8* locus was genetically modified to encode a C-terminal 13×myc epitope-tagged Zcf8 protein (Fig. 1A). The expression of the tagged gene remained under the control of the native promoter. We established that the activity of the tagged protein was only slightly diminished compared to the activity of the untagged version (Fig. S2A). Zcf8p constitutively localized in the *Candida* nucleus (Fig. S2B). Using stringent criteria to define segments enriched in the immunoprecipitated sample (see Materials and Methods), we identified 42 “peaks” or DNA regions bound by Zcf8p genome-wide (Fig. 1B; see also Table S1 in the supplemental material). The DNA sequences (500 nucleotides [nt] centered on the highest point of each peak) of the top scoring ∼1/4 of the bound regions were used to derive overrepresented DNA binding motifs *de novo*. The sequence 5′-TAAATCCG-3′ (Fig. 1C) was salient among the derived motifs because 5′-CCG-3′ is a well-established element of the sequences bound by other zinc cluster transcription regulators (15). We established that the derived sequence is indeed a bona fide binding site for Zcf8p because the purified recombinant protein bound a DNA fragment carrying this sequence in electrophoretic mobility shift assays (Fig. 1D). Furthermore, the introduction of point mutations in the 5′-AAA-3′ or 5′-CCG-3′ portion of the sequence impaired binding (Fig. 1D), although to different degrees (Fig. 1E).

10.1128/mBio.03020-21.2 FIG S2 Activity of tagged *ZCF8* allele, subcellular localization of Zcf8p, and transcriptional autoregulation. (A) Quantitative RT-PCR analysis comparing the activity of the tagged and untagged *ZCF8* alleles. Shown is the level of repression of *DAL52*, the top target of regulation of Zcf8p. The *DAL52* transcript levels measured in the homozygous deletion mutant (Δ*zcf8/*Δ*zcf8*) were used to estimate the percent repression achieved in each of the three indicated strains. The experimentally validated *TAF10* transcript (M.-A. Teste, M. Duquenne, J. M. François, J.-L. Parrou, BMC Mol Biol 10:99, 2009, https://doi.org/10.1186/1471-2199-10-99) served as reference control to normalize the quantitative reverse transcription-PCR (qRT-PCR) data. Shown are the means ± SD (*n *= 3). The Δ*zcf8*/*ZCF8*-MYC strain was used in the chromatin immunoprecipitation experiment. (B) Subcellular localization of GFP-tagged Zcf8p. Arrowheads indicate nuclei. Staining with FM4-64 delineates the vacuoles (marked with “v”). (C) Schematic of the reporter strains. In strain I (left), one *ZCF8* allele was replaced by *YFP*. In strain II, the second *ZCF8* allele was deleted. In both strains, the expression of the reporter is driven by the native *ZCF8* promoter. (D) YFP fluorescence emitted by the reporter strains described in panel C. Cells were grown overnight in YPD and diluted in PBS. Fluorescence was measured using a flow cytometer. Ten thousand cells were evaluated per strain per experiment. Plotted are the means ± SD of three independent experiments. The Student’s *t* test was used in the statistical analysis. Download FIG S2, TIF file, 0.9 MB. Copyright © 2021 Reuter-Weissenberger et al. 2021 Reuter-Weissenberger et al. https://creativecommons.org/licenses/by/4.0/ This content is distributed under the terms of the Creative Commons Attribution 4.0 International license.

10.1128/mBio.03020-21.5 TABLE S1 List of ChIP-Seq peaks and associated genes. This file includes a list of all Zcf8p ChIP peaks. The C. albicans chromosome and genome coordinates, fold enrichment as well as each peak’s nearest annotated ORF are indicated. Download Table S1, XLSX file, 0.02 MB. Copyright © 2021 Reuter-Weissenberger et al. 2021 Reuter-Weissenberger et al. https://creativecommons.org/licenses/by/4.0/ This content is distributed under the terms of the Creative Commons Attribution 4.0 International license.

Gene Ontology (GO) analyses of the ∼50 ORFs associated with Zcf8 ChIP “peaks” in their upstream intergenic region revealed generic terms such as “sequence-specific DNA binding” (*P* = 0.00055), “biofilm formation” (*P* = 0.00033), “cell aggregation” (*P* = 0.00033), “transmembrane transport” (*P* = 0.00575), and “regulation of filamentous growth” (*P* = 0.0074) overrepresented in the data set. About half of the ∼50 ORFs remain unannotated in the *Candida* Genome Database. We noticed that about a quarter of the unambiguously annotated Zcf8p-regulated ORFs had roles connected to either vacuole or intracellular vesicle trafficking: for example, the vacuolar basic amino acid transporter *VBA1* (16); the conserved oligomeric Golgi complex *COG2* (17, 18), an essential component of a cytosolic tethering complex that functions in protein trafficking to mediate fusion of transport vesicles to Golgi compartments; the unconventional SNARE in the endoplasmic reticulum *USE1* (19, 20), which is involved in retrograde traffic from the Golgi to the endoplasmic reticulum (ER); the putative oxidoreductase *C1_10840C* (21) which is involved in late endosome to Golgi transport; and *C4_03050C* which has homology to S. cerevisiae genes described as vacuole-related genes. These findings raised the possibility that Zcf8p may regulate some aspect of vacuole biology or intracellular trafficking in C. albicans.

### Zcf8p’s top targets of regulation localize to the fungal vacuole.

To further define the transcripts regulated by Zcf8p, we conducted a transcriptome (RNA-Seq) experiment in which we included three C. albicans strains: the *zcf8* deletion mutant, a strain overexpressing *ZCF8* (i.e., in this strain *ZCF8* expression is driven by the strong promoter *TDH3*) and the wild-type reference strain (Fig. 1F). This experiment identified 39 and 45 transcripts with significant changes in expression when comparing the wild type versus the deletion mutant (negative log_10_ *P* value > 10 and log_2_ fold change > |0.8|) or the wild type versus the *ZCF8* overexpression strain (negative log_10_ *P* value > 10 and log_2_ fold change > |1.5|), respectively (Fig. 1G and H and Table S2). As highlighted in Fig. 1G and H, a subset of transcripts downregulated in the *zcf8* deletion mutant were upregulated in the *ZCF8* overexpression strain and vice versa (e.g., *C4_05580C*, *CR_10320W*, and *DAL52*), as expected. The inclusion of the strain overexpressing *ZCF8*, nonetheless, expanded the list of putative *ZCF8*-regulated transcripts. GO searches with the entire list of differently expressed ORFs retrieved “amino acid biosynthesis” (*P* = 0.00193) and “amino acid metabolism” (*P* = 0.00927) as terms overrepresented in the data set.

10.1128/mBio.03020-21.6 TABLE S2 List of differentially expressed transcripts in *zcf8* deletion and *ZCF8* overexpression strains. This file includes a list of the C. albicans genes whose expression was up- or downregulated in the *zcf8* deletion mutant or in a strain overexpressing *ZCF8*. Download Table S2, XLSX file, 0.02 MB. Copyright © 2021 Reuter-Weissenberger et al. 2021 Reuter-Weissenberger et al. https://creativecommons.org/licenses/by/4.0/ This content is distributed under the terms of the Creative Commons Attribution 4.0 International license.

**FIG 2:**
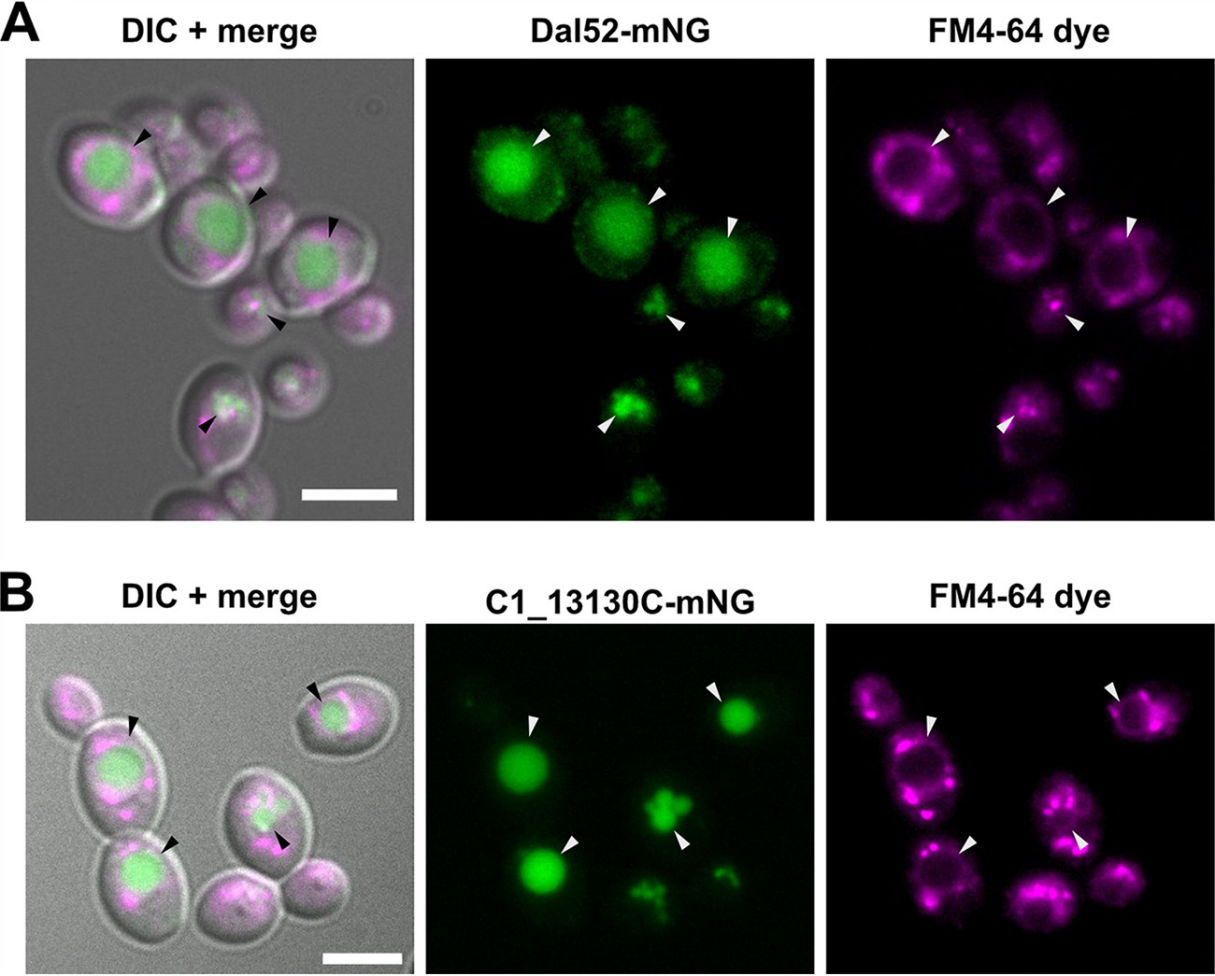
Putative allantoate and amino acid permeases regulated by Zcf8p are targeted to the fungal vacuole. (A and B) Subcellular localization of two gene products transcriptionally regulated by Zcf8p. The sequence encoding the C. albicans codon-optimized reporter mNeonGreen (mNG) was introduced in frame at the 3′ end of the *DAL52* (A) and *C1_13130C* (B) genes. The FM4-64 dye stains the fungal vacuole (indicated by arrowheads) and intracellular vesicles resulting from endocytosis. DIC, differential interference contrast. Bars, 5 μm.

Two of the top targets of Zcf8p regulation (which we define here as the genes associated with a Zcf8p ChIP peak in their 5′ intergenic region and displaying the largest change in RNA-Seq reads when comparing a deletion mutant versus the wild-type reference strain) were *DAL52*, which is annotated as “putative allantoate permease” and *VBA1*, which encodes a vacuolar basic amino acid transporter (Fig. 1B, I, and J). To establish the subcellular localization of the product encoded by *DAL52*, we fused this gene in its native chromosomal location to a *Candida*-optimized version of the fluorescent reporter mNeonGreen (22). Strikingly, Dal52p displayed intravacuolar localization (the vacuole is outlined with the lipophilic dye FM4-64) (Fig. 2A). A similar intravacuolar distribution pattern was observed for *C1_13130C* (Fig. 2B), another gene that we identified as a target of Zcf8p regulation in the ChIP-Seq experiment and remains annotated as “putative histidine permease.” These findings further hinted at a connection between Zcf8p and the fungal vacuole.

### Zcf8p functions primarily as a transcriptional repressor.

Our RNA-Seq data indicated that the genes under direct control of Zcf8p, i.e., the genes that in addition to showing a change in transcript levels also displayed a ChIP “peak” in their 5′ intergenic region, were for the most part subject to transcriptional repression by this protein (Fig. 1B). A common feature found in transcriptional regulatory circuits is that the regulator controls not only other target genes but also its own expression by binding to its promoter (15). Our ChIP data indicated that the Zcf8 protein also bound to the intergenic region upstream of the *ZCF8* ORF (Table S1), an arrangement that is suggestive of autoregulation. To test this model and determine whether Zcf8p exerted positive or negative regulation on itself, we constructed reporter strains in which one copy of the *ZCF8* ORF was replaced by yellow fluorescent protein (YFP) (Fig. S2C). The second *ZCF8* copy either remained intact or was deleted (Fig. S2C). The YFP levels (quantified by flow cytometry) were significantly lower in the heterozygous strain harboring an intact *ZCF8* copy compared to the isogenic *zcf8*Δ strain, which is consistent with Zcf8p repressing its own expression (Fig. S2D). While it cannot be ruled out that *ZCF8* may also act as an activator on a yet-to-be-determined set of genes, our results suggest that the Zcf8 protein acts primarily as a transcriptional repressor; as such, its autoregulation generates a negative-feedback loop.

### *zcf8* mutation and *ZCF8* overexpression result in altered vacuole properties.

**FIG 3:**
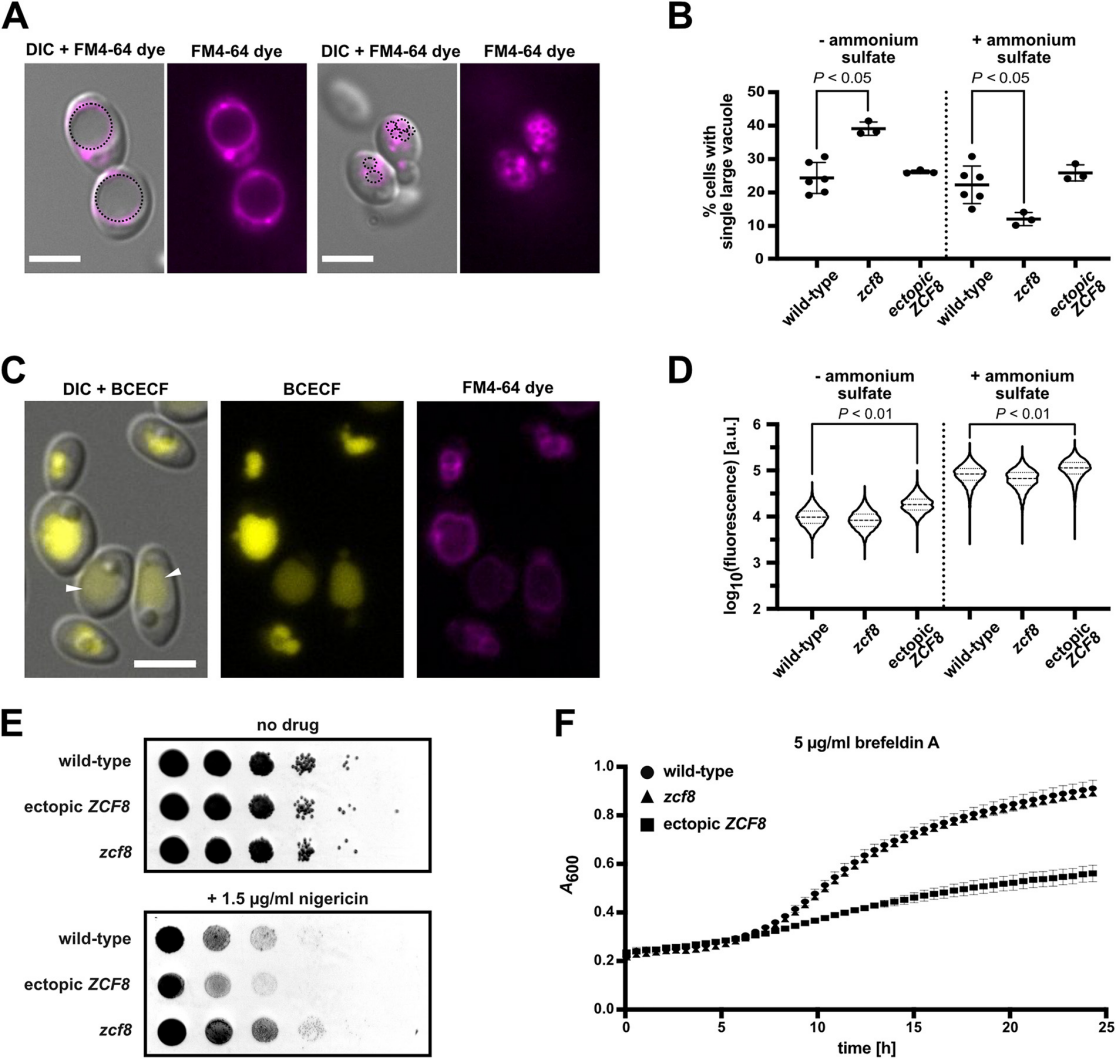
Zcf8p regulates vacuolar properties in C. albicans. (A and B) Vacuolar morphology in C. albicans cells is dependent on *ZCF8*. (A) The number of vacuoles within the fungal cell changes in response to conditions in the surroundings and can range from a single, large organelle to multiple, smaller compartments. (B) The fraction of cells (percentage) displaying a single large vacuole is plotted. The wild-type strain, *zcf8* deletion mutant, and a strain overexpressing *ZCF8* were evaluated. Cells were grown in the presence (+) and absence (−) of ammonium sulfate and stained using FM4-64 to visualize the vacuole(s). Each dot represents the mean of one experiment in which >500 cells were scored. The experiment was repeated at least three times. The Mann-Whitney U test was used in the statistical analysis. Bars, 5 μm. (C and D) Vacuolar pH increases in C. albicans cells overexpressing *ZCF8*. Cells were stained with the vacuolar pH indicator BCECF which emits higher fluorescence at higher pH. The microscopy images shown in panel C illustrate the variation in vacuolar fluorescence intensity (a proxy for variation in vacuolar pH) within a cell population. Arrowheads point to vacuoles with lower fluorescent signal (i.e., lower pH). (D) Quantification by flow cytometry of the fluorescence emitted by BCECF in single cells grown in media containing or lacking ammonium sulfate. Fluorescence is shown in arbitrary units. The median values and quartiles are indicated with dashed and dotted lines, respectively. Ten thousand cells were evaluated per strain in each individual experiment. Data shown are derived from at least four independent experiments. The Mann-Whitney U test was used in the statistical analysis. Bar, 5 μm. (E) Spot assay showing the increased resistance of the *zcf8* mutant and increased susceptibility of the strain overexpressing *ZCF8* to nigericin. Serially diluted cell suspensions were spotted on defined agar medium containing ammonium sulfate as the nitrogen source. Pictures were taken after 24 h of incubation. (F) Growth assay showing the susceptibility of the strain overexpressing *ZCF8* to brefeldin A. Cells were grown in defined liquid medium containing ammonium sulfate as the nitrogen source.

The fungal vacuole is a dynamic organelle. The presence of a single, large vacuole in yeast cells is thought to reflect nutrient limitation or other stress as these conditions induce vacuolar fusion events (11). Yet yeast cells can contain up to five vacuoles per cell (23) and the number can vary depending on extracellular and intracellular conditions (11). Because the presence of either a single large vacuole or multiple smaller compartments is a defining feature of the organelle, we first sought to evaluate this trait in Zcf8-deficient C. albicans cells and in a strain overproducing the regulator. We evaluated C. albicans cells grown in defined medium lacking any nitrogen source or supplemented with ammonium sulfate. The reasons for the choice of nitrogen as a variable in the growth conditions were twofold. First, our genome-wide ChIP and RNA-Seq data pointed to (intra)cellular transporters of amino acids or other nitrogen-rich molecules (e.g., allantoate) as top targets of Zcf8p’s regulation (Fig. 1 and 2). Second, the importance of mobilizing amino acids in and out of the vacuole is particularly evident when cells face nitrogen-poor conditions (24, 25). To visualize the vacuoles, we stained C. albicans cells with the lipophilic dye FM4-64 (Fig. 3A). As shown in Fig. 3B, the fraction of cells displaying a single large vacuole differed significantly in the *zcf8* mutant compared to the wild-type reference strain: in the presence of ammonium sulfate, the *zcf8* mutant had fewer single-vacuole cells, whereas under nitrogen starvation the fraction of single-vacuole cells increased almost threefold. These results suggest that *ZCF8* is required for C. albicans to withstand large variations in vacuolar fusion and fission events due to changes in nitrogen availability.

Fungal vacuoles are acidic compartments. The acid pH is not only a defining feature of these organelles but can also be regulated and is important for vacuolar functions such as transport of ion and metabolites, fusion and fission events, and protein sorting (26). Thus, we sought to establish whether the vacuoles of Zcf8-deficient C. albicans cells or of a strain overproducing the regulator display alterations in pH. As described in the previous paragraph, we evaluated C. albicans cells grown in defined medium lacking any nitrogen source or supplemented with ammonium sulfate. The pH-sensitive fluorescent indicator BCECF [2′,7′‐bis‐(2‐carboxyethyl)‐5‐(and ‐6)‐carboxyfluorescein] was used to indirectly assess the pH within the vacuolar lumen (Fig. 3C). The fluorescence intensity emitted by this compound upon excitation is proportional to the pH in the respective compartment, within the pH range ∼6 to ∼9 (higher fluorescence intensity, more alkaline pH) (27, 28). We used flow cytometry to quantify the fluorescence in each population of cells, and the observed distributions are shown in Fig. 3D. This analysis revealed that *ZCF8* overexpression resulted in cells containing vacuoles of more alkaline pH, particularly when the cells were grown in medium lacking ammonium sulfate. The *zcf8* deletion mutant, on the other hand, displayed a trend toward vacuoles of more acidic pH. The pH or acidity of the vacuole, therefore, changes in response to alterations in *ZCF8* expression.

We found that neither autophagy nor endocytosis, two cellular processes associated with the vacuole, are altered to a significant extent in the *zcf8* mutant strain. Green fluorescent protein (GFP)-Atg8, a well-established marker of autophagy, was employed to assay bulk autophagy by monitoring vacuolar delivery of GFP-Atg8 by Western blotting analysis (Fig. S3) (29). The uptake of the fluorescent dye FM4-64 was used to visualize membrane internalization and endocytic delivery to the vacuole (Fig. S4) (30).

10.1128/mBio.03020-21.3 FIG S3 *ZCF8* does not influence autophagy. (A) Western blotting of the autophagy marker GFP-Atg8p in wild-type background and in the *zcf8* deletion mutant using an anti-GFP antibody. These two strains, together with the untagged control, were grown in defined liquid medium without amino acids and ammonium sulfate for 3 or 6 h. The accumulation of free GFP (due to Atg8p degradation) is a proxy for autophagy. Blotting with an anti-tubulin 1 antibody shows equal loading. (B) Vacuolar delivery of mNeonGreen-Atg8p by bulk autophagy visualized by fluorescence microscopy. Autophagosome is marked with white arrowhead. Bar, 5 μm. (C) Quantification of microscopy images shown in panel B. Plotted is the fraction of cells with a visible autophagosome as a proxy for autophagic activity. Every dot represents the percentage of cells per microscope field (only fields with >20 cells were scored). Twenty frames were scored in each experiment. Plotted are the results from five independent experiments. Download FIG S3, TIF file, 0.9 MB. Copyright © 2021 Reuter-Weissenberger et al. 2021 Reuter-Weissenberger et al. https://creativecommons.org/licenses/by/4.0/ This content is distributed under the terms of the Creative Commons Attribution 4.0 International license.

10.1128/mBio.03020-21.4 FIG S4 Evaluating the role of ZCF8 in endocytosis. (A) Internalization of endocytic vacuoles in C. albicans. Cells grown in defined liquid medium without amino acids and ammonium sulfate were incubated with the lipophilic dye FM4-64 for 10 or 20 min, washed with ice-cold PBS, and kept on ice until evaluated by fluorescence microscopy. Representative images are shown. Bars, 5 μm. (B) Quantification of images shown in panel A. Plotted is the mean fluorescence (± SD) emitted by the internalized FM4-64 dye. The fluorescence emitted by each cell was measured using FIJI. At least 400 cells (derived from >20 microscope fields) were measured per strain per time point per experiment. Data collected from four independent experiments are shown. Download FIG S4, TIF file, 1.9 MB. Copyright © 2021 Reuter-Weissenberger et al. 2021 Reuter-Weissenberger et al. https://creativecommons.org/licenses/by/4.0/ This content is distributed under the terms of the Creative Commons Attribution 4.0 International license.

Consistent with the observed alterations in vacuolar properties, we established that deletion of *ZCF8* rendered C. albicans resistant to nigericin, a K^+^/H^+^ ionophore that targets, among other organelles, the fungal vacuole (31), whereas overexpression resulted in hypersensitivity to the drug (Fig. 3E). The ionophore introduces ion “channels” into membranes; as a consequence, it breaks down the pH gradient between cytoplasm and vacuole disrupting the organelle’s function. The *ZCF8* overexpression strain also displayed increased sensitivity to brefeldin A, a drug that targets the Golgi and impairs vesicle formation and transport (Fig. 3F). Collectively, the findings described in this section demonstrate that key properties of the fungal vacuole such as number and pH are controlled by Zcf8p in C. albicans.

### C. albicans upregulates amino acid transporters in response to nigericin.

Because the *ZCF8* gene is connected to C. albicans susceptibility/resistance to nigericin, we were interested in establishing more broadly the response of the fungus to this drug. To do so, we conducted a RNA-Seq analysis comparing nigericin-treated versus untreated C. albicans cultures. To minimize secondary effects caused by drug treatment, we evaluated cells after 30 min of incubation with nigericin. To our knowledge, there is no published data set on the response of any yeast to this drug at the transcriptome level.

**FIG 4:**
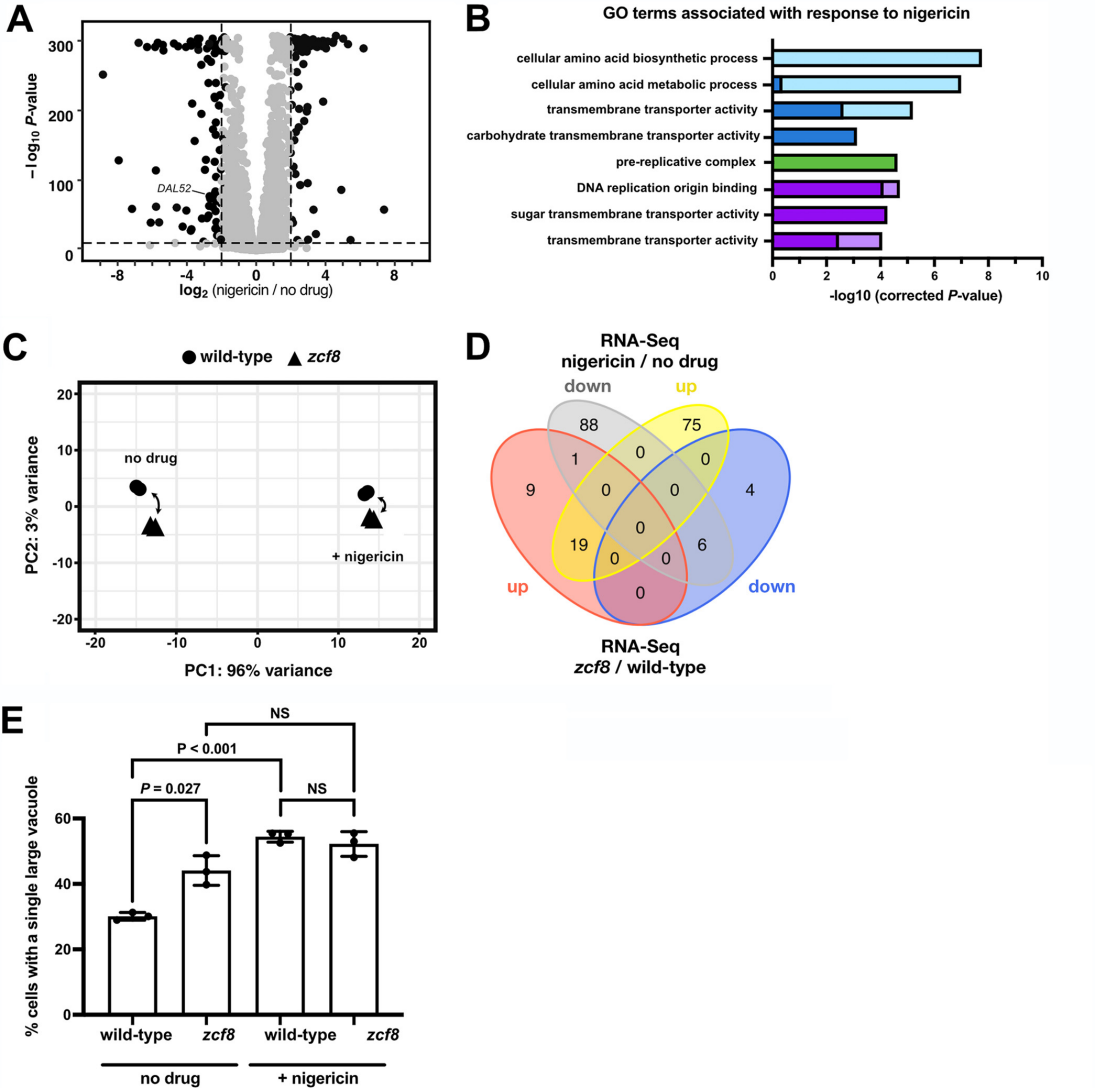
C. albicans upregulates amino acid transporters in response to nigericin. (A) Transcripts up- or downregulated in response to nigericin treatment. Wild-type cells were grown in medium without amino acids and ammonium sulfate and treated for 30 min with the drug (at a final concentration of 15 μg/ml). Shown is a volcano plot where each dot represents one transcript. Significantly up- or downregulated transcripts are shown in black. (B) GO terms enriched in the set of transcripts that changed their expression level in response to nigericin. Light and dark colors indicate enrichment in the set of drug-induced or drug-repressed genes, respectively. Blue, green, and purple indicate “process”, “function,” or “component” categories. (C) Principal-component analysis based on results of RNA-Seq experiment conducted with the wild-type strain and *zcf8* deletion mutant in the presence and absence of nigericin. (D) Venn diagram showing the overlap between the set of nigericin-regulated genes and up- or downregulated transcripts following *ZCF8* deletion. (E) Altered vacuole morphology in response to nigericin. Cells cultured under nitrogen starvation conditions were treated with the drug (15 μg/ml) for 30 min and washed twice with PBS before staining with FM4-64. Plotted is the percentage of cells displaying a single large vacuole. Bars represent the means ± standard deviations (SD) (error bars) of three independent experiments in which >150 cells were scored per strain per condition. The Student’s *t* test was used in the statistical analysis. NS, not significant.

Our RNA-Seq experiment identified 188 protein-coding transcripts with altered expression in response to the drug (using the following cutoffs: −log_10_ *P* > 10 and expression changes >2-fold; Fig. 4A and Table S3). GO term searches of processes, functions, and components identified “cellular amino acid biosynthetic process” and “cellular amino acid metabolic process” as the top categories enriched in the set of nigericin-induced genes, whereas “prereplicative complex” was the top term overrepresented in the set of drug-repressed genes (Fig. 4B). An overall comparison of the responses to the drug at the transcriptome level between the wild-type reference strain and *zcf8* mutant can be visualized in the principal-component analysis (PCA) shown in Fig. 4C. While the drug itself explains a substantial portion (96%) of the variance in this experiment, the wild type and *zcf8* mutant are placed closer to each other in the presence of nigericin (compared to the samples derived from cultures without the drug). The distance that separates two samples in the PCA plot are proportional to their similarity/differences; therefore, this analysis indicates that, at a global level, the transcriptional changes effected by *ZCF8* are diminished in the presence of the drug. Furthermore, the Venn diagram showing the overlap between nigericin- and *ZCF8*-regulated genes (Fig. 4D) indicates that more than half of the upregulated transcripts in the *ZCF8* deletion mutant (19 of 29 transcripts) were also upregulated in response to the drug.

10.1128/mBio.03020-21.7 TABLE S3 List of differentially expressed transcripts in response to nigericin. This file includes a list of the C. albicans genes whose expression was significantly altered in response to nigericin treatment in the wild-type reference strain and the *zcf8* deletion mutant. Download Table S3, XLSX file, 0.03 MB. Copyright © 2021 Reuter-Weissenberger et al. 2021 Reuter-Weissenberger et al. https://creativecommons.org/licenses/by/4.0/ This content is distributed under the terms of the Creative Commons Attribution 4.0 International license.

Consistent with the findings in the transcriptome data, we established that nigericin altered the fraction of *Candida* cells displaying a large single vacuole or multiple smaller organelles (Fig. 4E). Of importance to this report, the drug had no significant effect in the *zcf8* mutant, further supporting a connection between the vacuole and regulator.

### Zcf8p modulates *Candida* interactions with host epithelial cells in a vacuole-dependent manner.

**FIG 5:**
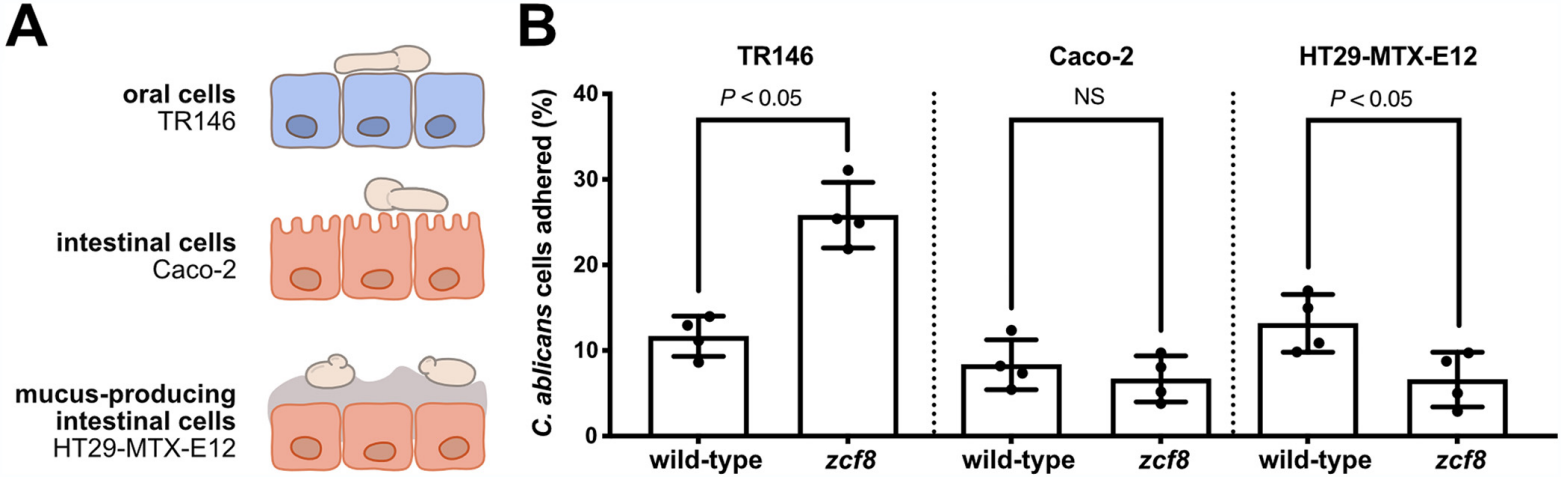
Zcf8p modulates *Candida* adherence to host epithelial cells. (A) Diagram illustrating the three epithelial cell systems employed to probe interactions with C. albicans cells. (B) Adhesion of C. albicans to intestinal and oral epithelial cells. Host cells were coincubated with C. albicans wild‐type or *zcf8* deletion mutant for 1 h. After extensive washing, cells were fixed and stained. Adhered cells were visualized in a fluorescence microscope and quantified in 50 fields for each replicate. Each black circle represents the percentage of adhered cells in one of four independent experiments. Bars represent the means ± SD. The Mann-Whitney U test was used in the statistical analysis.

In previous studies, we had established that the C. albicans *zcf8* null mutant strain displays two striking opposing phenotypes in mice: (i) impaired gut colonization after oral gavage (4) and (ii) increased fungal load in tongues in the oropharyngeal candidiasis model (5). Because Finkel et al. (8) had reported that the *zcf8* mutant shows reduced fungal adherence to silicone, we hypothesized that the mouse-associated phenotypes may also be due to alterations in the attachment of the deletion mutant strain to mucosal surfaces. To test this idea, we probed the attachment of the fungus to intestinal (Caco-2 cells and the mucus-producing HT29-MTX-E12 cell line) and oral epithelial (TR146) cells (Fig. 5A). The inclusion of a mucus-secreting intestinal cell line follows the observation that, in the murine colon, C. albicans cells do not directly contact epithelial cells but rather remain adjacent to, or immersed within, the intestinal mucous layer (4, 32). We found that, compared to the wild-type reference strain, the isogenic *zcf8* mutant adhered significantly less to the mucus-producing intestinal cells (Fig. 5B). Strikingly, the same mutant displayed increased adherence to the oral epithelial cells (Fig. 5B). These findings are consistent with, and provide a basis to, the opposing phenotypes reported in mice (impaired gut colonization on the one hand and higher fungal load in oral infection on the other hand).

**FIG 6:**
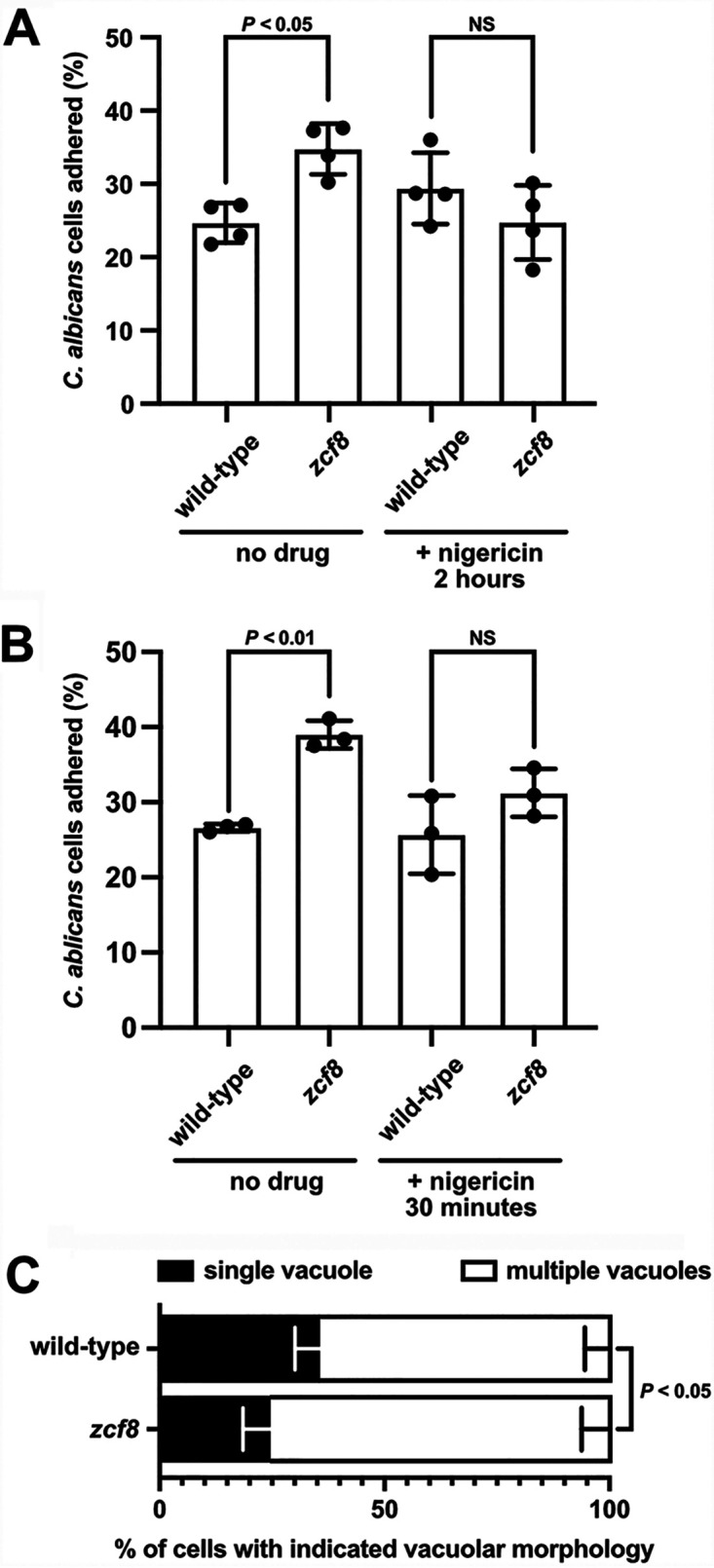
Increased adherence of *zcf8* mutant cells to oral epithelial cells requires intact vacuole function. (A and B) C. albicans adhesion to oral epithelial cells. *Candida* cultures (wild‐type strain or *zcf8* deletion mutant) were treated with the drug nigericin (at a final concentration of 15 μg/ml) for 2 h (A) or 30 min (B) (or left untreated in the control experiment). The drug was eliminated by washing twice the fungal cells with PBS before adding *Candida* to the oral epithelial cells. Adherence was monitored as described in the legend to Fig. 5B. Each black circle represents the percentage of adhered cells in one of four independent experiments. Bars represent the means ± SD. The Mann-Whitney U test was used in the statistical analysis. (C) C. albicans vacuolar morphology upon attachment to host epithelial cells. *Candida* cells (wild‐type strain or *zcf8* deletion mutant) were added to oral epithelial cells and incubated for 1 h. Nonadhered fungal cells were washed away. Vacuoles in the adhered *Candida* population were visualized by FM4-64 staining. Plotted is the percentage of cells displaying the indicated vacuolar morphology. Bars represent the means ± SD of five independent experiments. At least 30 cells were evaluated per strain per replicate. The Mann-Whitney U test was used in the statistical analysis.

The results described so far in this report link Zcf8p to two traits: on the one hand, altered attachment to mucosal surfaces (Fig. 5) and, on the other hand, regulation of the fungal vacuole (Fig. 3). A logical question, therefore, is whether the two traits are linked. To probe whether impairing vacuolar function has any consequence on fungal adherence, we treated *Candida* cells with the drug nigericin and, after washing away the drug, brought *Candida* and oral epithelial cells together to quantify fungal adhesion. As shown in Fig. 6A and B, the enhanced adherence caused by the *zcf8* mutation was abolished in the presence of the drug. Furthermore, we found that the vacuole number differed between wild-type and mutant strains upon contact with host epithelial cells (Fig. 6C). There is, therefore, good correlation between Zcf8’s effects on fungal adhesion and vacuolar function.

### *ZCF8* is a negative regulator of *Candida* filamentation in host tissues.

**FIG 7:**
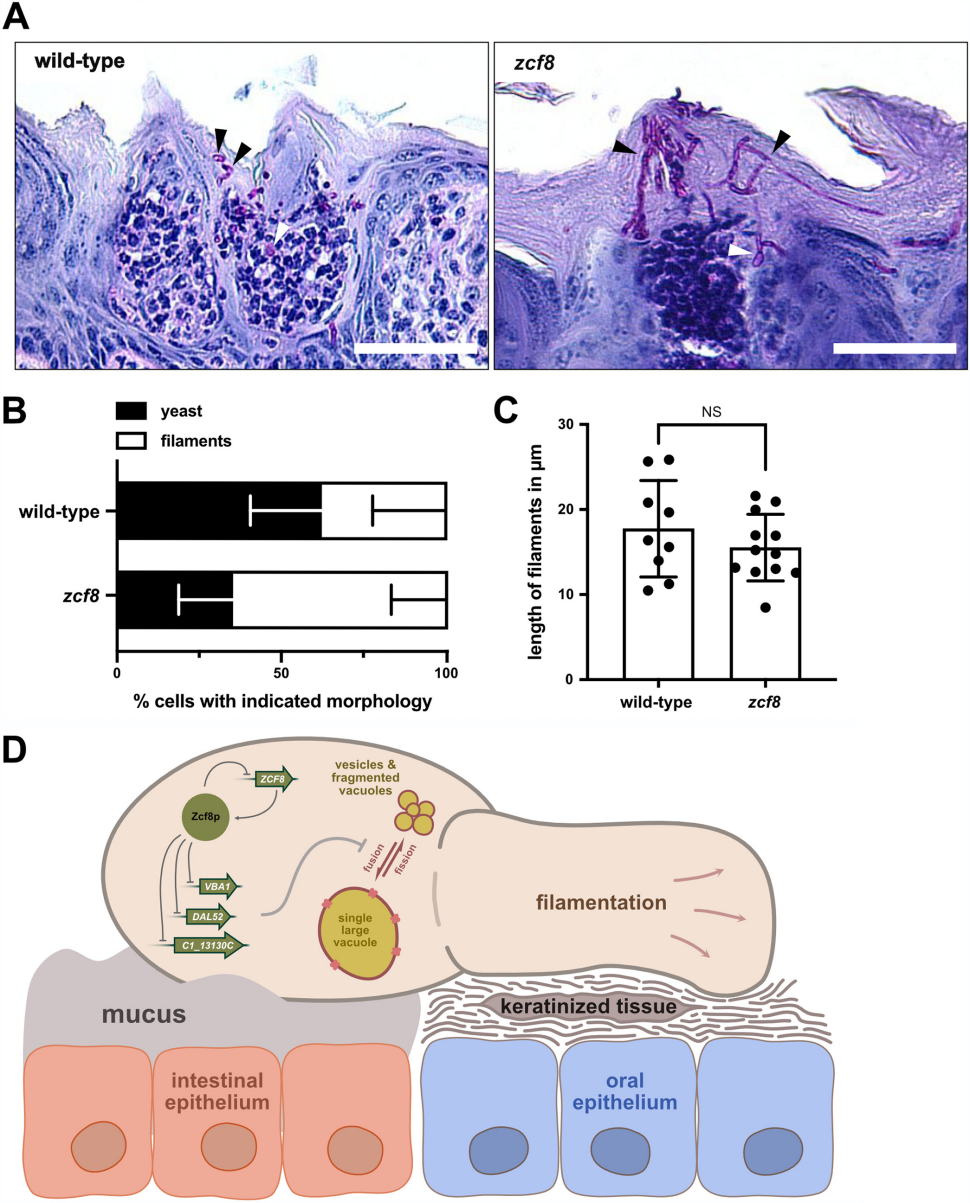
*ZCF8* is a negative regulator of *Candida* filamentation in host tissue. (A) Shown are representative images of sites along the tongue where C. albicans established infection. PAS‐stained sagittal sections were prepared from tongues 72 h after infection with either the wild‐type reference strain (left) or the *zcf8* deletion mutant. Bars, 100 μm. (B) Percentage of fungal cells found in the murine tongue in the oval-shaped “yeast” form or the filamentous form. Cells were considered filamentous when the length of the fungal cell exceeded by at least 2 times its diameter. At least 35 fungal cells were evaluated per strain per mouse (*n* = 4 animals). Bars represent the means ± SD. The Mann-Whitney U test was used in the statistical analysis. (C) Length of C. albicans filamenting cells in infected tongues. Clearly distinguishable single filamenting cells in PAS-stained sections were randomly chosen and their length measured. Each dot represents one cell. One hundred fifty cells were evaluated per strain. Bars represent means ± SD. The Mann-Whitney U test was used in the statistical analysis. (D) Model depicting how Zcf8p-regulated products targeted to the vacuole impinge upon the morphology of the organelle (i.e., presence of a single large vacuole or smaller, fragmented compartments) and subsequently on *Candida* attachment to mucosal surfaces. The yeast form of the fungus attaches better to intestinal mucus (4), whereas the filamentous form adheres more efficiently to oral cells (5).

Mutations in genes that encode major structural components of the vacuole often lead to defects in C. albicans filamentation (12, 33). Thus, we sought to investigate whether the *zcf8* mutant cells displayed any alteration in morphology during colonization of the murine host. We evaluated mouse tongue sections prepared from animals infected with either wild-type *Candida* or the isogenic *zcf8* mutant strain (Fig. 7A). Quantification of the oval-shaped “yeast” cells as well as filaments in the tissues showed an increased proportion of the latter morphology in the tongues infected with the *zcf8* mutant (Fig. 7A and B). The average length of the filaments did not vary between both strains (Fig. 7C). The results of this experiment indicate that *ZCF8* is a negative regulator of C. albicans filamentation in host tissues.

## DISCUSSION

In this report, we have investigated the molecular function of a previously uncharacterized fungal gene which is involved in C. albicans colonization of the murine oral cavity and intestinal tract. We establish that this gene, *ZCF8*, encodes a bona fide transcription regulator that governs multiple vacuole-associated traits in this fungus and modulates *Candida* interactions with mammalian epithelial cells. Several lines of evidence support this notion. (i) Zcf8p’s top targets of transcriptional regulation encode proteins that localize to the vacuole (Fig. 1 and 2). (ii) The number of vacuoles per fungal cell and the pH of the organelle depended on *ZCF8* (Fig. 3A to D). (iii) Deletion or overexpression of *ZCF8* altered the resistance/sensibility of the fungus to nigericin and brefeldin A, two drugs that disrupt vacuolar function and vesicle formation/transport, respectively (Fig. 3E and F). (iv) *ZCF8* modulated the adherence of *Candida* cells to epithelial cells (Fig. 5). (v) Vacuole fragmentation (*in vitro* and *in vivo*) is correlated with the regulator’s effect on fungal adhesion (Fig. 6). On the basis of these findings, we posit that *Candida* employs Zcf8p to maintain vacuole homeostasis and that such activity impinges upon the physical contacts that fungal cells establish with mucosal surfaces (Fig. 7D).

The overlap between the Zcf8p ChIP and transcriptome data sets is rather low (Fig. 1I and J). Several reasons, some of them concerning the methodology, could explain this. For example, the laboratory culture conditions may not have been ideal to promote binding of the Zcf8 protein to some target promoters, particularly those with relatively low occupancy. Also, the thresholds that we used to call peaks was rather high; such strategy minimizes false-positive results (our main goal) but some true signal is lost as well. Beyond these technical considerations, it is also plausible that to promote or repress transcription on certain promoters, Zcf8p must act together with other unidentified transcription factors. In the absence of conditions that promote the activity of these additional factors, no expression changes would be observed. It is important to point out, however, that the main finding of our study, i.e., the discovery of Zcf8 as a regulator of the fungal vacuole, relies for the most part on the characterization of target genes for which we observed both Zcf8p binding and transcript changes (Fig. 1B).

A major function of the fungal vacuole is the storage and recycling of amino acids (12). The importance of mobilizing amino acids in and out of the vacuole is particularly evident when cells face nitrogen-poor environments (24, 25). Under these conditions, cells use amino acids produced by autophagic degradation of proteins in the vacuole. As illustrated in Fig. 7D, we have identified multiple transporters of amino acids or nitrogen-rich molecules (e.g., allantoate) as targets of Zcf8p regulation: for example, *VBA1*, a major vacuolar transporter of amino acids, and *DAL52*, a putative allantoate transporter that localizes to the vacuole (Fig. 2). Whether these transporters operate by moving their substrates into, or out of, the vacuole (or are bidirectional) remains to be established. Our results, however, imply that the misregulation of these transporters (and/or related products) can lead to significant vacuolar fusion and fission events in response to extracellular nitrogen availability (Fig. 3B). In agreement with this idea, it has been shown that deletion of *VBA4*, a nonessential transporter localized to the S. cerevisiae vacuole, can result in significant changes to the number of vacuoles within the yeast cell (34). *ZCF8*, therefore, appears to play a key role in limiting these events and, thus, preventing large fluctuations in the number of vacuoles per cell (i.e., maintaining vacuole homeostasis) (Fig. 7D). Further studies are needed, however, to establish whether *ZCF8* governs nitrogen and/or amino acid metabolism in addition to the described vacuole-associated phenotypes.

How does the regulation of products targeted to the vacuole impinge upon attachment to mucosal surfaces? We propose that the misregulation of vacuole-associated products leads to alterations in the morphology and cell surface of the fungus, which ultimately dictate the attachment of the fungal cell to host surfaces (Fig. 7D). In support of this idea, it has been demonstrated that mutations in genes that encode major structural components of the vacuole often lead to defects in C. albicans filamentation (12, 33). Furthermore, a well-established response to nitrogen starvation in C. albicans is filamentation (35–37). We found a larger proportion of filamenting cells in host tissues infected with the *zcf8* mutant strain (Fig. 7A and B), indicating that *ZCF8* modulates this trait *in vivo*. Our ChIP-Seq data revealed that Zcf8p bound upstream of several well-established regulators of yeast-to-filament transition in C. albicans (e.g., *BRG1* and *RFG1*). Thus, it is plausible that *ZCF8*’s effects on morphology are in part mediated by altering the expression of those regulators (although we found no evidence of changes in transcript levels in any of those ORFs in our RNA-Seq experiments). As a negative regulator of filamentation, *ZCF8* is expected to have contrasting effects on C. albicans attachment to intestinal surfaces versus oral mucosa. That is because filaments bind more efficiently to keratinocytes (5), whereas the round yeast form attaches better to mucus (4) (Fig. 7D). Our results indeed support this prediction.

Mutations affecting vacuole function, biogenesis, and inheritance often lead to defects in fungal virulence (12, 38). The reports that have established a connection between the vacuole and *Candida* virulence, however, have relied almost exclusively on mutations that eliminate core structural components of the organelle such as the vacuolar proton-translocating ATPase (V-ATPase). The V-ATPase is a multisubunit complex that generates a proton gradient which is the driving force for the transport of metabolites into the vacuole and for the maintenance of the low pH in the vacuolar lumen and other endocytic vesicles (39). Loss of C. albicans V-ATPase activity alkalinizes the vacuolar lumen and has pleiotropic effects, including pH-dependent growth, calcium sensitivity, and cold sensitivity (14). Depletion of one of the V-ATPase subunits, *VMA3*, leads to alkalinization of the lumen, abnormal vacuole morphology, suppressed filamentation, and attenuated macrophage killing (14). Null mutations in *VMA7*, a different subunit, also resulted in defective vacuole acidification, reduced growth at alkaline pH and sensitivity toward the presence of metal ions (40). The *vma7* null mutant strain was also avirulent in a mouse model of systemic candidiasis (40). The results described in this report establish that several properties of the *Candida* vacuole are under regulation of the Zcf8 protein and that mutations in this regulator (rather than in structural components of the vacuole) can also result in alterations of the fungus-host interplay.

While multiple protein kinases are known to regulate vacuolar functions, only a few transcription regulator proteins have been shown thus far to impinge upon the functioning of this organelle. One of these proteins is the transcriptional repressor Mot3p in S. cerevisiae (41). Mot3 represses transcription of genes needed for ergosterol biosynthesis. Ergosterol is required for endocytosis and homotypic vacuole fusion, providing a link between Mot3 and these processes. Indeed, *mot3* mutant cells display a number of related phenotypes, including abnormal vacuolar morphology and poor growth on alkaline media (41). The *MOT3* ortholog in C. albicans is termed *CAS5* (*C4_01190W*) and has been reported to regulate cell wall stability and cell cycle dynamics in this species (42). The S. cerevisiae Forkhead box proteins Fkh1p and Fkh2p may represent another example, as they have been shown to bind upstream of the *VMA6* and *VMA8* genes, which encode subunits of the vacuolar ATPase (43). C. albicans harbors a single Forkhead box protein, Fkh2p, which does not appear to regulate transcription of any *VMA* gene. The *Candida* Fkh2p rather promotes the expression of hypha-specific and pathogenesis-related genes (44, 45). Another regulatory protein that has been linked to the fungal vacuole is Ume6p (46). In S. cerevisiae, Ume6p negatively regulates autophagy (47, 48), a process tightly linked to the function of the fungal vacuole. In C. albicans, Ume6p promotes hyphal formation, and its ectopic expression has been shown to stimulate the generation of vacuolated subapical compartments in the filamenting cells (49). Yet it remains to be established whether Ume6p directly controls the expression of any vacuolar component in *Candida*. While it may seem counterintuitive that an apparently important function for fungi in general (i.e., vacuole homeostasis) be under the control of different transcription regulators across species, the “rewiring” of transcriptional networks, at least in ascomycete yeasts, seems to be rather common (50). Indeed, it has been documented that key biological processes such as yeast mating, meiosis, and biofilm formation have undergone extensive modifications in their regulation over evolutionary timescales (51–53).

Finally, our finding that *ZCF8* promotes *Candida* attachment to intestinal mucus but weakens binding to keratinocytes may explain the rather contradictory phenotypes that had been reported for this gene in mice. On the one hand, the *zcf8* mutant had been shown to be impaired in gut colonization. The reduced attachment of the mutant strain to intestinal mucus could account for this phenotype. On the other hand, the same mutation caused higher fungal loads in tongues in a murine OPC model, which correlates well with the enhanced binding of the *zcf8* mutant to oral cells. Our results, therefore, illustrate how divergent outcomes in the fungus-host interplay can be traced back to the function of a single regulatory gene.

## MATERIALS AND METHODS

### Strains and growth conditions.

The C. albicans strains used in this study are derivatives of clinical isolate SC5314 (54) and are listed in Table S4 in the supplemental material. Oligonucleotides and plasmids are listed in Tables S5 and S6, respectively. Gene deletions were carried out as described previously (55, 56). YFP-, GFP-, mNeonGreen- and MYC-tagging strategies for C. albicans have been described (6, 57). The *TDH3* promoter-driven overexpression strains were generated using the plasmids and procedures described before (58). C. albicans strains were routinely grown at 30°C in yeast-peptone-dextrose (YPD) medium. When defined medium was employed, cells from overnight cultures were collected by centrifugation, washed twice with phosphate-buffered saline (PBS), diluted 1:10 in nitrogen starvation medium (BD Difco YNB without amino acids and ammonium sulfate, supplemented with 2% glucose) or medium containing ammonium sulfate as the only nitrogen source (DB Difco YNB without amino acids, supplemented with 2% glucose), and incubated for 6 h at 30°C with shaking at 220 rpm. Nigericin (catalog no. SML1779; Merck, Germany) and brefeldin A (catalog no. B6542; Merck, Germany) were used at a final concentration of 1.5 μg/ml or 5 μg/ml, respectively (unless otherwise indicated).

10.1128/mBio.03020-21.8 TABLE S4 List of Candida albicans strains used in this study. Download Table S4, PDF file, 0.2 MB. Copyright © 2021 Reuter-Weissenberger et al. 2021 Reuter-Weissenberger et al. https://creativecommons.org/licenses/by/4.0/ This content is distributed under the terms of the Creative Commons Attribution 4.0 International license.

10.1128/mBio.03020-21.9 TABLE S5 List of oligonucleotides used in this study. Download Table S5, PDF file, 0.1 MB. Copyright © 2021 Reuter-Weissenberger et al. 2021 Reuter-Weissenberger et al. https://creativecommons.org/licenses/by/4.0/ This content is distributed under the terms of the Creative Commons Attribution 4.0 International license.

10.1128/mBio.03020-21.10 TABLE S6 List of plasmids used in this study. Download Table S6, PDF file, 0.1 MB. Copyright © 2021 Reuter-Weissenberger et al. 2021 Reuter-Weissenberger et al. https://creativecommons.org/licenses/by/4.0/ This content is distributed under the terms of the Creative Commons Attribution 4.0 International license.

### Chromatin immunoprecipitation followed by deep sequencing (ChIP-Seq).

The *ZCF8*-MYC-tagged strain along with an untagged control were grown in YPD broth overnight at 30°C. The chromatin immunoprecipitation procedure was carried out as described previously (6, 57). Processing of raw data was conducted following the procedures outlined in other report (59).

### Peak calling and DNA motif analysis.

The R package “bPeaks” (v.1.2) (60) was employed to detect significant peaks using an untagged immunoprecipitation (IP) as control. Based on the selected parameters (T1: 2, T2: 1, T3: 1.8, T4: 0.9), the program spotted 148 regions as potential peaks. We visualized all these peaks in MochiView (v.1.46) (61) and refined the list based on the following criteria. (i) Only peaks located in intergenic regions were maintained. (ii) Peaks adjacent to highly expressed genes were removed because these places tend to bind to almost all DNA binding proteins nonspecifically (6, 62–64). (iii) Only peaks that were consistent in at least two of four replicates were kept. This curation resulted in 42 peaks representing 51 genes (some peaks lie in divergently transcribed intergenic regions). To derive *de novo* putative DNA binding motifs, 500-nt sequences centered on the midpoint of the top ∼1/4 of scoring peaks were fed into the Regulatory Sequence Analysis Tools (RSAT) for fungal organisms (http://rsat.sb-roscoff.fr). The utility ran on default parameters with the background model set for C. albicans.

### Recombinant protein purification and electrophoretic mobility gel shift assay.

The putative DNA binding domain of Zcf8p (amino acids 57 to 158) was amplified by PCR using oligonucleotides JCP2014 and JCP2026 and C. albicans genomic DNA (gDNA) as the template. The PCR fragment was subsequently digested and ligated into the SmaI/XhoI sites of plasmid pLIC-H3 (65). This plasmid is designed to overproduce 6×His-tagged proteins in Escherichia coli. The E. coli strain BL21 was used as the host of the expression plasmid. Cells were grown to an optical density at 600 nm (OD_600_) of ∼0.8, and expression was induced with 0.5 mM isopropyl-β-d-thiogalactopyranoside (IPTG). After ∼3-h induction, cells were pelleted and stored at −80°C. Cells were lysed by sonication. The His-tagged protein was affinity purified from the lysate using nickel-nitrilotriacetic acid (Ni-NTA) agarose beads (Qiagen, Netherlands). Amicon Ultra-15 centrifugal filters (Merck) were used to exchange buffer and concentrate the protein. Protein concentration was estimated in Rothi-blue (Carl Roth, Germany)-stained gels using known amounts of bovine serum albumin as standards. Gel shift assays were carried out as described in reference 66.

### Transcriptome analyses (RNA-Seq).

Cells harvested from overnight cultures in YPD were washed three times with PBS and diluted 1:10 in 3 ml yeast nitrogen base (YNB) without amino acids and ammonium sulfate. After 5.5 h of incubation at 30°C in a shaker, nigericin (at a final concentration of 15 μg/ml) or an equal volume of dimethyl sulfoxide (DMSO)/ethanol solution (mock) were added and incubated for an additional 30 min. Cells were collected by centrifugation. RNA was prepared using the RiboPure kit for yeast (Thermo Fisher Scientific, Waltham, USA). Illumina library preparation and strand-specific sequencing were carried out by Novogene (Cambridge, UK) following standard operating procedures. We obtained 41 to 48 million reads per sample (paired-end, 150-nt reads). Read processing, quality control, mapping, and differential gene expression analyses followed standard computational procedures described by our laboratory (59, 67). Briefly, Illumina adaptor sequences were removed using the Trimmomatic tool (v.0.39) (68). Trimmed reads were aligned to the C. albicans reference genome assembly 21 (www.candidagenome.org) using STAR (v.2.7.3a) (69). Count files were generated using HTSeq (v.0.11.2) (70). Raw read counts were loaded into R and analyzed with the DESeq2 package (v.1.28.1) (71), and volcano plots were created using the R package EnhancedVolcano (v.1.6.0) (https://github.com/kevinblighe/EnhancedVolcano). Two biological replicates were included in each RNA-Seq analysis.

### Microscopy analyses.

Live-cell imaging of fungal cells at high resolution was performed using a wide field epifluorescence microscopy (Leica, DMI 6000 B). A 63× oil plan fluor objective (numerical aperture, 1.4) was employed. Excitation at 520 nm was used to visualize the subcellular localization of the mNeonGreen-tagged proteins. Excitation at 440 nm was used to visualize BCECF (18 μM) in the vacuolar lumen, and excitation at 550 nm was employed to detect vesicle/vacuolar membranes with FM4-64 (10 μM). Images were processed using FIJI (v.2.1.0).

### Drug sensitivity assay.

Cells grown overnight in YPD liquid medium were washed with PBS, and serially diluted cell suspensions (initial cell concentration, 5 × 10^8^ cells/ml) were spotted onto YNB agar containing ammonium sulfate and nigericin at a final concentration of 1.5 μg/ml (2 μM).

### Flow cytometry analysis.

Cells were stained with 18 μM BCECF for 30 min at 30°C as established in reference 28 and analyzed using the BD Accuri C6 flow cytometer and the appurtenant software (v.1.0.264.21). The R package “flowCore” (v.1.11.20) was used to extract event-level information from the flow cytometry standard (FCS) files, and subsequent data manipulation and visualization were done using GraphPad (v.9.1.0).

### Epithelial cell culture.

The human oral buccal cell line TR146 (Sigma-Aldrich, St. Louis, MO, USA) was cultured in Dulbecco modified Eagle medium (DMEM)/F12 medium, supplemented with 10% fetal bovine serum (FBS). The human Caucasian colon adenocarcinoma cell line Caco-2 (kindly provided by Cynthia Sharma, Institute for Molecular Infection Biology [IMIB], Würzburg, Germany) was cultivated in DMEM supplemented with 10% FBS. HT29-MTX-E12 (Sigma-Aldrich, St. Louis, MO, USA) were seeded on transwell membranes (Corning Transwell 0.4-μm-pore-size polycarbonate membrane; 12-mm inserts purchased from Sigma-Aldrich, Germany) and cultured in DMEM supplemented with 10% FBS, 5 mM glutamine, and 5 mM nonessential amino acids (Thermo Fisher Scientific) and allowed to grow for 21 days with regular medium exchange twice per week. All cell lines were cultivated in a humidified incubator at 37°C and 5% CO_2_.

### Adherence assays.

Attachment of C. albicans to epithelial cells was measured either through fluorescence microscopy as described earlier for TR146 cells (5), which was also used for the Caco-2 cell line, or plated in the case of the HT29-MTX-E12 cell line. Briefly, cells of all three epithelial cell lines were challenged with C. albicans cells at a multiplicity of infection (MOI) of 1. After 1 h, cells were washed three times with PBS to remove nonadherent C. albicans cells. TR146 and Caco-2 cells were fixed with 4% paraformaldehyde and incubated overnight with a primary anti-*Candida* antibody (Invitrogen, Carlsbad, CA, USA). On the next day, cells were washed and stained with a secondary Alexa Fluor 594-conjugated goat anti-rabbit antibody (Life Technologies, Carlsbad, USA). Imaging was done using a Leica DMI 6000B microscope (Leica, Wetzlar, Germany). The number of adhered fungal cells was acquired in 50 frames per strain. *Candida* adherence to mucus covering the HT29-MTX-E12 transwells was measured by scraping all cells from wells (after extensive washing to remove nonadhered cells). Cells were pelleted, plated on YPD plates, and incubated for 48 h at 30°C. Adherence was determined in four independent experiments for each cell line.

C. albicans cells treated with nigericin were also assayed for attachment to the oral epithelial cells TR146. Briefly, fungal cells were incubated with nigericin or a mock solution (DMSO/ethanol [EtOH]) for 2 h at 30°C, washed twice, and added to TR146 cells. After the addition of *Candida*, cells were incubated for 2 h at 37°C with 5% CO_2_. Afterwards, cells were washed with PBS (to remove nonadhered cells) and fixed, stained, and quantified as described above.

### OPC infection model.

The animal experiments were approved by the local government of Lower Franconia (Regierung von Unterfranken), Germany (protocol number 55.2-2531.01-50/14). Murine oral infections were conducted as described previously (5). Briefly, the C. albicans inoculum for infection was prepared using a 1-ml overnight culture in YPD, washed twice with sterile PBS, and serially diluted to a final concentration of 5 × 10^6^ cells/ml. A sterile cotton ball (2.5 mg) was soaked for 1 h in 100 μl of this suspension. Six- to 9-week-old female C57BL/6J mice (Charles River, Sulzfeld, Germany) were anaesthetized with a mixture of ketamine (100 mg/kg of body weight; Zoetis, NJ, USA) and xylazine (6 mg/kg; CP-Pharma, Burgdorf, Germany) in sterile PBS which was injected intraperitoneally. A cotton ball saturated with C. albicans cells was placed sublingually for 90 min. Animal weight was monitored on a daily basis. Three days after inoculation, mice were euthanized, and tongues were removed, split sagittally, and fixed for histology.

### Histology and PAS staining.

Mouse tongues were removed and split sagittally. Fixation of one half was done overnight in 10% buffered formalin (10% of a 37% formaldehyde solution, 0.65% Na_2_HPO_4_, and 0.4% NaH_2_PO_4_) and then washed several times with PBS. The tissue was embedded in paraffin on a paraffin embedding machine using a standard program consisting of washing steps in increasing ethanol concentrations, xylol baths, and 3 h in melted paraffin at 62°C. The resulting blocks were stored at room temperature. For microscopy, 4-μm sections were prepared and stained following standard periodic acid-Schiff (PAS) staining protocols. Fungal cells clearly distinguishable in PAS-stained sections were scored as filamenting if their length was >2 times their diameter (5).

### Gene Ontology.

The Gene Ontology term finder tool of the *Candida* Genome Database (www.candidagenome.org) was used to identify overrepresented terms in the data sets.

### Data availability.

The ChIP-Seq and RNA-Seq data generated in this study have been deposited in the Gene Expression Omnibus (GEO) database (accession numbers GSE181652, GSE181653, and GSE181655).
