# Supplementary figures and images for: A Fungal Transcription Regulator of Vacuolar Function Modulates Candida albicans Interactions with Host Epithelial Cells
Source: mBio. 2021 Nov 16;12(6):e03020-21. doi: 10.1128/mBio.03020-21 (PMC8593675; doi:10.1128/mBio.03020-21)

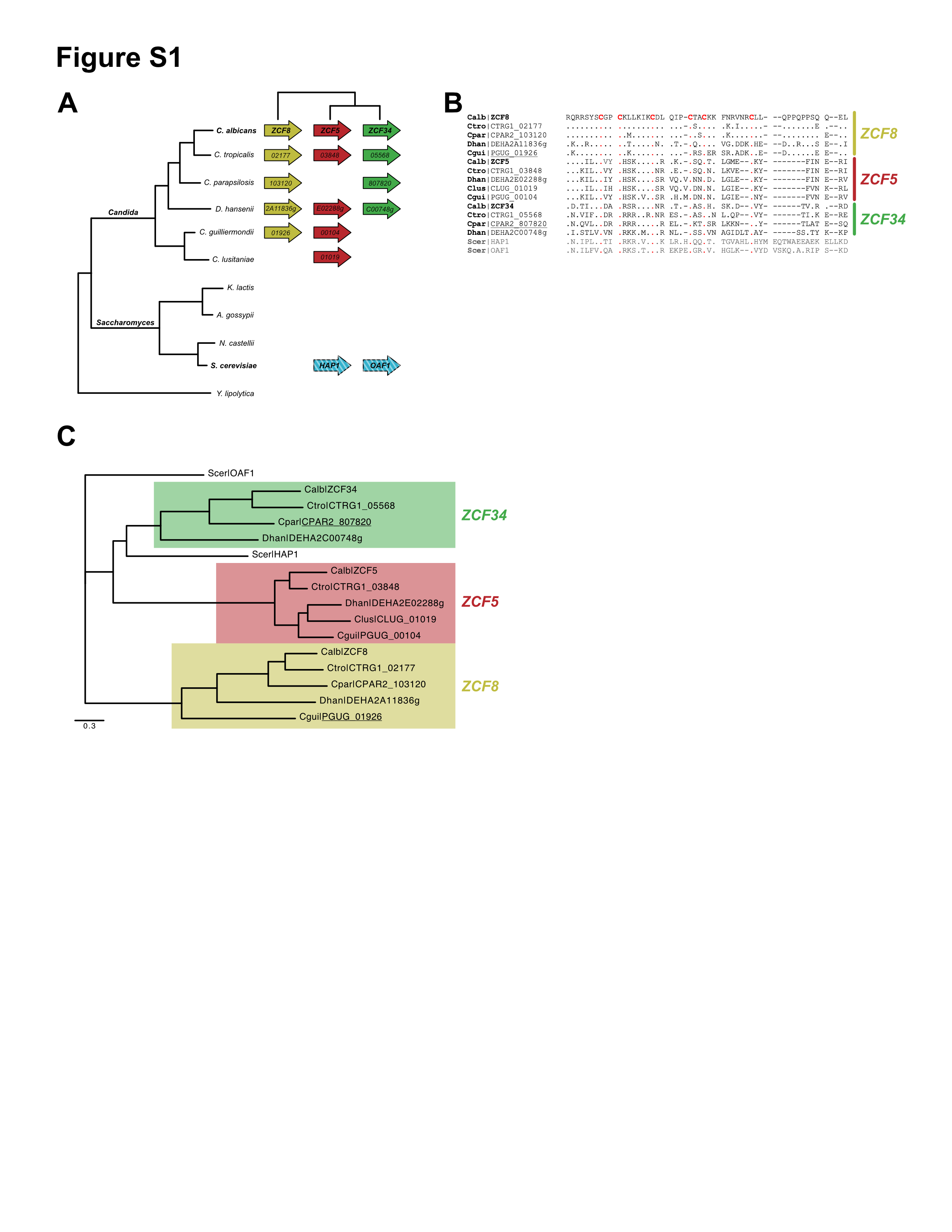

Supplement: FIG S1 [file mbio.03020-21-sf001.tif]

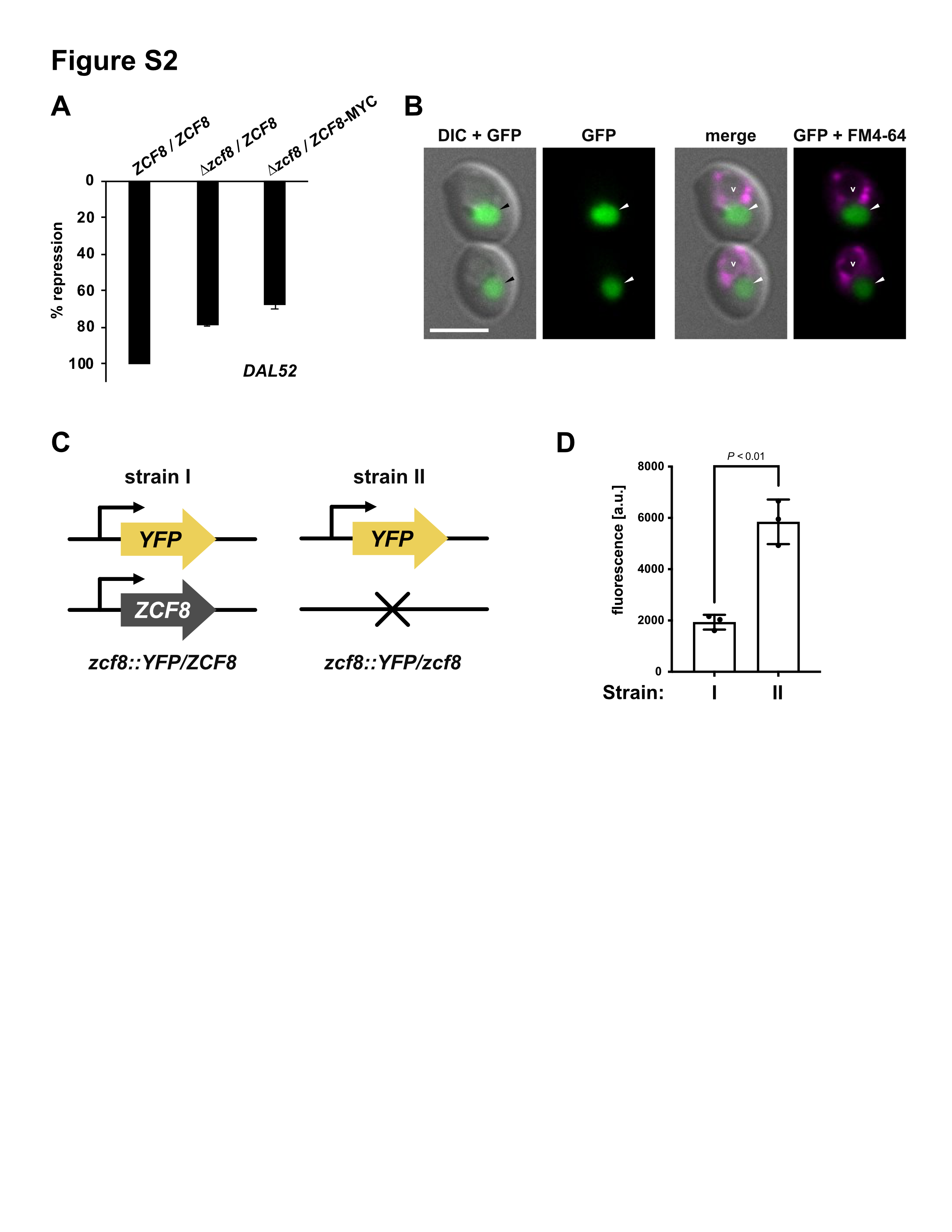

Supplement: FIG S2 [file mbio.03020-21-sf002.tif]

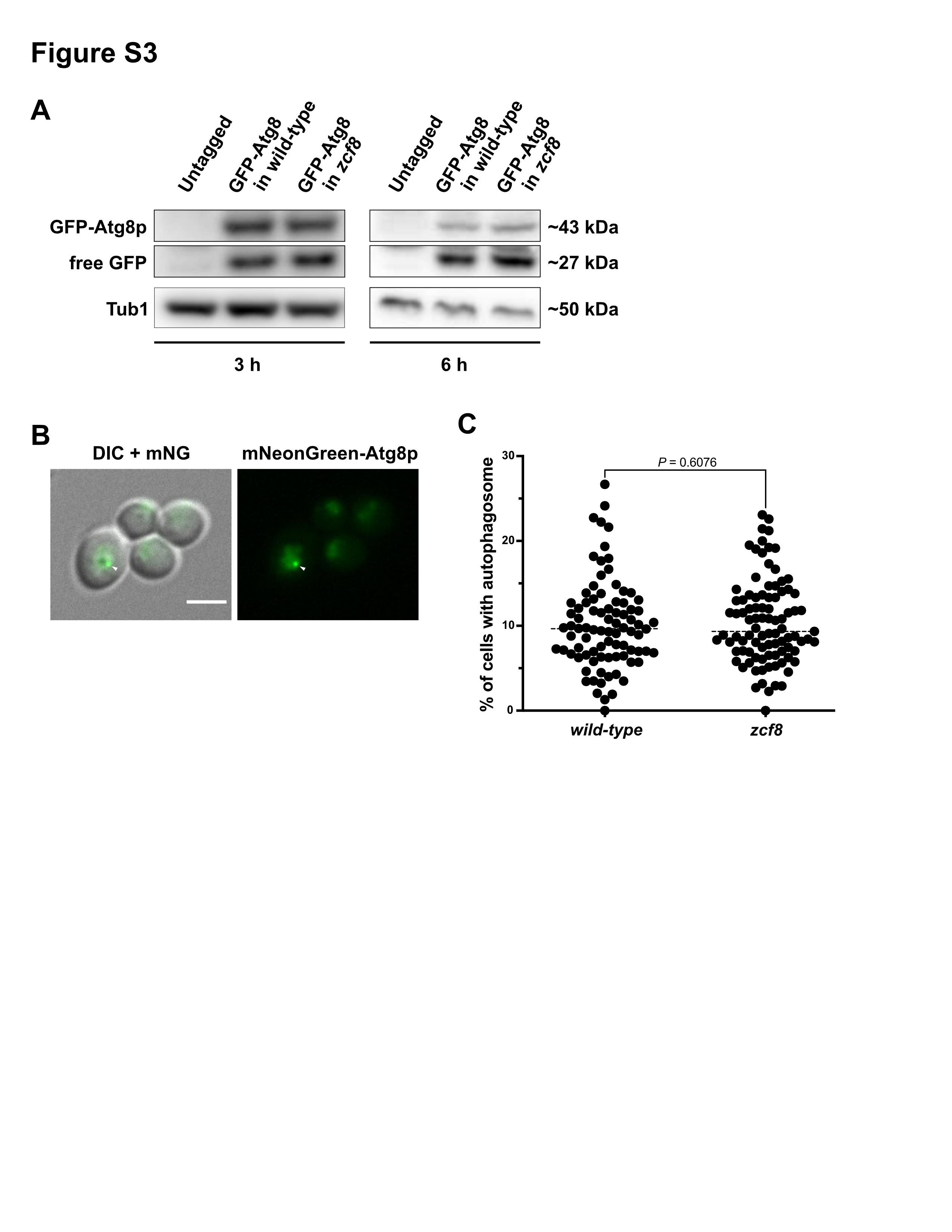

Supplement: FIG S3 [file mbio.03020-21-sf003.tif]

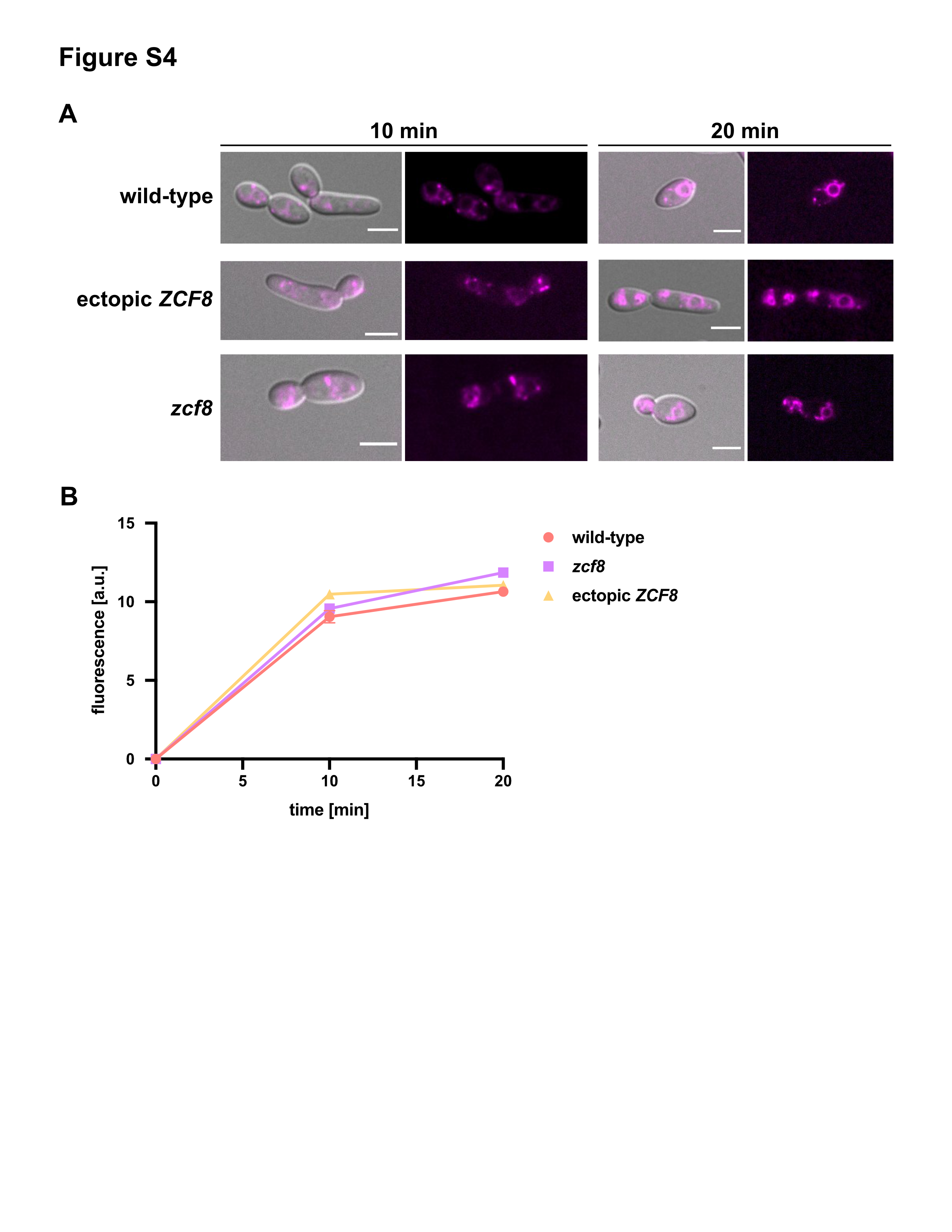

Supplement: FIG S4 [file mbio.03020-21-sf004.tif]
