# Supplementary material for: A Fungal Transcription Regulator of Vacuolar Function Modulates Candida albicans Interactions with Host Epithelial Cells
Source: mBio. 2021 Nov 16;12(6):e03020-21. doi: 10.1128/mBio.03020-21 (PMC8593675; doi:10.1128/mBio.03020-21)
Supplement: TABLE S4 [file mbio.03020-21-st004.pdf]

Table S4. *Candida albicans* strains used in this study.

Name	Parent	Genotype	Source
JCP_398	SN152	$\frac{ura3\Delta::\lambda imm434::URA3-IRO1}{ura3\Delta::\lambda imm434}\frac{arg4::hisG}{arg4::hisG}\frac{his1::hisG}{his1::hisG}\frac{leu2::hisG}{leu2::hisG}$	Noble and Johnson *et al.*, 2005
JCP_738	SN152	$\frac{ura3\Delta::\lambda imm434::URA3-IRO1}{ura3\Delta::\lambda imm434}\frac{arg4::hisG}{arg4::hisG}\frac{his1::hisG}{his1::hisG}\frac{leu2::hisG}{leu2::hisG}\frac{zcf8\Delta::CdHIS1}{zcf8\Delta::CmLEU2}$	Homann *et al.*, 2009
JCP_796	SN152	$\frac{ura3\Delta::\lambda imm434::URA3-IRO1}{ura3\Delta::\lambda imm434}\frac{arg4::hisG}{arg4::hisG}\frac{his1::hisG}{his1::hisG}\frac{leu2::hisG}{leu2::hisG}\frac{zcf8\Delta::YFP}{ZCF8}$	This study
JCP_936	SN152	$\frac{ura3\Delta::\lambda imm434::URA3-IRO1}{ura3\Delta::\lambda imm434}\frac{arg4::hisG}{arg4::hisG}\frac{his1::hisG}{his1::hisG}\frac{leu2::hisG}{leu2::hisG}\frac{zcf8\Delta::YFP}{zcf8\Delta::CmLEU2}$	This study
JCP_942	SN250	$\frac{ura3\Delta::\lambda imm434::URA3-IRO1}{ura3\Delta::\lambda imm434}\frac{arg4::hisG}{arg4::hisG}\frac{his1::hisG}{his1::hisG}\frac{leu2::hisG::CdHIS1}{leu2::hisG::CmLEU2}\frac{ZCF8-13xMYC}{zcf8\Delta}$	This study
JCP_520	SC5314		Gillum *et al.*, 1984
JCP_1072	SC5314	$\frac{TDH3p-YFP-ZCF8}{zcf8\Delta}$	This study
JCP_1102	SC5314	$\frac{zcf8\Delta}{zcf8\Delta}$	This study
JCP_1139	SC5314	$\frac{C\_13130C-mNeonGreen}{C\_13130C}$	This study
JCP_1140	SC5314	$\frac{DAL52-mNeonGreen}{DAL52}$	This study

References

Gillum, A. M., Tsay, E. Y. H., & Kirsch, D. R. 1984. Isolation of the *Candida albicans* gene for orotidine-5_′-phosphate decarboxylase by complementation of *S. cerevisiae* ura3 and *E. coli* pyrF mutations. Mol Gen Genet 198:179–182.

Homann, O. R., Dea, J., Noble, S. M., & Johnson, A. D. 2009. A phenotypic profile of the *Candida albicans* regulatory network. PLoS Genet 5:e1000783.

Noble, S. M., & Johnson, A. D. 2005. Strains and strategies for large-scale gene deletion studies of the diploid human fungal pathogen *Candida albicans*. Eukaryot Cell 4:298–309.
