# Supplementary material for: A Fungal Transcription Regulator of Vacuolar Function Modulates Candida albicans Interactions with Host Epithelial Cells
Source: mBio. 2021 Nov 16;12(6):e03020-21. doi: 10.1128/mBio.03020-21 (PMC8593675; doi:10.1128/mBio.03020-21)
Supplement: TABLE S5 [file mbio.03020-21-st005.pdf]

Table S5. Oligos used in this study.

Number	Description	Sequence (5'- 3')
ZCF8 deletion using pSFS2a		
JCP_2162	KpnI upstream *ZCF8* F	AGACTTGGTACCCGTGGCTGTATTGATGGATTGT
JCP_2163	XhoI upstream *ZCF8* R	AGATTACTCGAGAGAAGTTTGTGGCTGTTGGA
JCP_2164	NotI downstream *ZCF8* F	AGACGCGGCCGCTGCAAGATCAAACCCTTAGTCT
JCP_2165	SacII downstream *ZCF8* R	AGCTAGCCGCGGACGAGTGTTGTGTTGAAAGGT
ZCF8 ectopic expression (inserting *TDH3* promoter upstream *ZCF8*)		
JCP_1563	pCJN542 F	TTTCTTATATATATATTTTCTATTTAATTTATACTGGAATACATCAAGATTGATTGGAGATTGAATTGGCATCAAGCTTGCCTCGTCCCC
JCP_1478	pCJN542 R	GAATAATTCTTCACCTTTAGACATATTTGAATTCAATTGTGATG
JCP_1479	pAJ2181 YFP F	ATGTCTAAAGGTGAAGAATTATTC
JCP_1564	pAJ2181 YFP R	GGGTAGCACTCGATGAAGGTGAAGATGGTGAAGGCGAAGGTGATTCAGTATTAGATAGATTACTTTCCATATTTGAATTCAATTGTGATG
Substitution of *ZCF8* by *YFP* and *ZCF8* deletion		
JCP_1913	pAJ2182 YFP F	TATTTACTTTTCAATTCTTCCTTCCTTTTTCCCTGTCAAACTACATTCAACCTTAATTTTCATTCAAATATGTCTAAAGGTGAATTATTC
JCP_1387	pAJ2182 YFP R	CAAAACCAGATTTCCAGATTTCCAGTTATTTGTACAATTCATCCATACC
JCP_1388	pMBL179 SAT1 F	CTGGAAATCTGGAAATCTGGTTTTG
JCP_1914	pMBL179 SAT1 R	AATGATATTAACACTATTCTATCTATCTATATATATATCAATACAATTTACCCATATGTTTCCCAAATTAGGCGTCATCCTGTGCTCCCG
JCP_2172	*ZCF8* del F	CGTGGCTGTATTGATGGATTGT
JCP_2175	*ZCF8* del R	ACGAGTGTTGTGTTGAAAGGT
C-terminal tagging of *ZCF8* with 13xMyc		
JCP_1569	pADH34 13xMyc F	ATTTAAATTTGATGAAAATGTCGTTAATGAATATTTCAAACATTGTGTTGAACCAAATATCTTTGAACGGATCCCCGGGTTAATTAACGG
JCP_1570	pADH34 13xMyc R	TGATATTAACACTATTCTATCTATCTATATATATATCAATACAATTTACCCATATGTTTCCCAAGGCGGCCGCTCTAGAACTAGTGGATC
Tagging *C1_13130C* and *DAL52* with mNeonGreen		
JCP_2981	C1_13130C KpnI F	ATATGGTACCCTATTGGTGGAGATTATGGAG
JCP_2982	C1_13130C mNG R	CCTTTAGAAACCATACCACTACCACCGCACCAGAAATTGACAAATC
JCP_2983	*C1_13130C* pRB895 mNG F	GATTTGTCAATTTCTGGTGCGGTGGTAGTGGTATGGTTTCTAAAGG
JCP_2980	pRB895 mNG XhoI R	ATTACTCGAGCTATTTATACAATTCATCCATACC
JCP_2984	*C1_13130C* NotI F	TAATGCGGCCGCCATGATAAGAAATTTGTACG
JCP_2985	*C1_13130C* SacII R	TTATCCGCGGGTTGTTGTCTCTTTGTTCTG
JCP_2986	*DAL52* KpnI F	TATAGGTACCTGTTTCTTCAAATGTTGC
JCP_2987	*DAL52* mNG R	CCTTTAGAAACCATACCACTACCACCGAGACTATATCTAAAATTCAAATTTTC
JCP_2988	*DAL52* pRB895 mNG F	GAAAATTTGAATTTTAGATATAGTCTCGGTGGTAGTGGTATGGTTTCTAAAGG
JCP_2989	*DAL52* NotI F	ATATGCGGCCGCATGAGATGATGAGTTATGTTG

JCP_2990	*DAL52* SacII R	TTTACCGCGGTGGATACAGATATTGATTCC
Inserting *ZCF8* DNA binding domain sequence into pLIC-H3		
JCP_2024	SmaI *ZCF8* DBD F	AGCAGCCCCGGGACAACAAAACCTAAACGTCAAAG
JCP_2026	XhoI *ZCF8* DBD R	GTGGTGCTCGAGTTACTTTTCATAATTCTTCGAAGTAG
