# Supplementary material for: A Fungal Transcription Regulator of Vacuolar Function Modulates Candida albicans Interactions with Host Epithelial Cells
Source: mBio. 2021 Nov 16;12(6):e03020-21. doi: 10.1128/mBio.03020-21 (PMC8593675; doi:10.1128/mBio.03020-21)
Supplement: TABLE S6 [file mbio.03020-21-st006.pdf]

Table S6. Plasmids used in this study.

Number	Name	Description	Reference
JCP_054	pCJN542	source of *C. albicans TDH3* promoter	Nobile *et al.,* 2008
JCP_002	pSFS2a	*SAT1*-flipper	Reuss *et al.,* 2004
JCP_1118	pRB895	source of *C. albicans* optimized mNeonGreen	Fraser *et al.,* 2019
JCP_129	pMBL179	source of *NAT1* coding sequence	Dalal *et al.*, 2016
JCP_182	pAJ2182	source of *C. albicans* optimized YFP	Gerami-Nejad *et al.*, 2012
JCP_080	pADH34	source of *C. albicans* optimized 13xMYC tag	Hernday *et al.,* 2010
JCP_233	pLIC-H3	produce recombinant N-terminal 6-His tagged proteins in *E. coli*, upon IPTG-induction	Cain *et al*., 2012
JCP_809	pLIC-H3-*ZCF8*(DBD)	recombinant 6His-Zcf8p (DBD) production	This study
JCP_938	pSFS2a + upstream *ZCF8* + downstream *ZCF8*	used to replace *ZCF8* with *SAT1* flip recombinase cassette	This study
JCP_1132	pSFS2a + *DAL52*-mNeonGreen + downstream *DAL52*	used to tag *DAL52* with C-terminal mNeonGreen	This study
JCP_1133	pSFS2a + *C1_13130C* -mNeonGreen + downstream *C1_13130C*	used to tag *C1_13130C* with C-terminal mNeonGreen	This study

References

Cain, C. W., Lohse, M. B., Homann, O. R., Sil, A., & Johnson, A. D. 2012. A conserved transcriptional regulator governs fungal morphology in widely diverged species. Genetics 190:511–521.

Dalal, C. K., Zuleta, I. A., Mitchell, K. F., Andes, D. R., El-Samad, H., & Johnson, A. D. 2016. Transcriptional rewiring over evolutionary timescales changes quantitative and qualitative properties of gene expression. eLife 5:e18981.

Frazer, C., Hernday, A. D., & Bennett, R. J. 2019. Monitoring Phenotypic Switching in *Candida albicans* and the Use of Next-Gen Fluorescence Reporters. Curr Protoc Microbiol 53:1–27.

Gerami-Nejad, M., Forche, A., Mcclellan, M., & Berman, J. 2012. Analysis of protein function in clinical *C. albicans* isolates. Yeast, 29:303–309.

Hernday, A. D., Noble, S. M., Mitrovich, Q. M., & Johnson, A. D. 2010. Genetics and molecular biology in *Candida albicans*. Methods Enzymol 470:737–758.

Nobile CJ, Solis N, Myers CL, Fay AJ, Deneault JS, Nantel A, Mitchell AP, Filler SG. 2008. *Candida albicans* transcription factor Rim101 mediates pathogenic interactions through cell wall functions. Cell Microbiol 10:2180–2196.

Reuß, O., Vik, Å., Kolter, R., & Morschhäuser, J. 2004. The *SAT1* flipper, an optimized tool for gene disruption in *Candida albicans*. Gene 341:119–127.
